# Behavioral and Neural Correlates of Speech Motor Sequence Learning in Stuttering and Neurotypical Speakers: An fMRI Investigation

**DOI:** 10.1162/nol_a_00027

**Published:** 2021-02-01

**Authors:** Matthew Masapollo, Jennifer A. Segawa, Deryk S. Beal, Jason A. Tourville, Alfonso Nieto-Castañón, Matthias Heyne, Saul A. Frankford, Frank H. Guenther

**Affiliations:** Department of Speech, Language, and Hearing Sciences, Boston University, Boston, MA, USA; Department of Speech, Language, and Hearing Sciences, University of Florida, Gainesville, FL, USA; Department of Speech, Language, and Hearing Sciences, Boston University, Boston, MA, USA; Departments of Neuroscience and Biology, Stonehill College, Easton, MA, USA; Department of Speech, Language, and Hearing Sciences, Boston University, Boston, MA, USA; Department of Speech-Language Pathology, University of Toronto, Toronto, Canada; Department of Speech, Language, and Hearing Sciences, Boston University, Boston, MA, USA; Department of Speech, Language, and Hearing Sciences, Boston University, Boston, MA, USA; Department of Speech, Language, and Hearing Sciences, Boston University, Boston, MA, USA; Department of Speech, Language, and Hearing Sciences, Boston University, Boston, MA, USA; Department of Speech, Language, and Hearing Sciences, Boston University, Boston, MA, USA; Departments of Neuroscience and Biology, Stonehill College, Easton, MA, USA; Department of Biomedical Engineering, Boston University, Boston, MA, USA; Picower Institute for Learning and Memory, Massachusetts Institute of Technology, Cambridge, MA, USA

**Keywords:** speech motor control, motor sequence learning, stuttering, GODIVA model, fMRI

## Abstract

Stuttering is a neurodevelopmental disorder characterized by impaired production of coordinated articulatory movements needed for fluent speech. It is currently unknown whether these abnormal production characteristics reflect disruptions to brain mechanisms underlying the acquisition and/or execution of speech motor sequences. To dissociate learning and control processes, we used a motor sequence learning paradigm to examine the behavioral and neural correlates of learning to produce novel phoneme sequences in adults who stutter (AWS) and neurotypical controls. Participants intensively practiced producing pseudowords containing non-native consonant clusters (e.g., “GVAZF”) over two days. The behavioral results indicated that although the two experimental groups showed comparable learning trajectories, AWS performed significantly worse on the task prior to and after speech motor practice. Using functional magnetic resonance imaging (fMRI), the authors compared brain activity during articulation of the practiced words and a set of novel pseudowords (matched in phonetic complexity). FMRI analyses revealed no differences between AWS and controls in cortical or subcortical regions; both groups showed comparable increases in activation in left-lateralized brain areas implicated in phonological working memory and speech motor planning during production of the novel sequences compared to the practiced sequences. Moreover, activation in left-lateralized basal ganglia sites was negatively correlated with in-scanner mean disfluency in AWS. Collectively, these findings demonstrate that AWS exhibit no deficit in constructing new speech motor sequences but do show impaired execution of these sequences before and after they have been acquired and consolidated.

## INTRODUCTION

Stuttering is a neurodevelopmental disorder affecting children into adulthood with devastating social effects that impede speech communication. The presenting clinical signs of the disorder include involuntary repetitions and prolongations of phonemes, syllables, or words, as well as involuntary silent pauses (Bloodstein & Ratner, 2008). Despite considerable research, researchers still have a relatively poor understanding of the neural deficits underlying the disorder (see Craig-McQuaide et al., 2014, for a review). Neurobiological accounts of stuttering, such as Alm (2004), Max et al. (2004), and recently Connally et al. (2018) and Chang and Guenther (2020), proposed that stuttering disfluencies arise from an underlying defect in the (left-lateralized) cortico-basal-ganglia-thalamocortical (cortico-BG) loops (Alexander et al., 1986), which are hypothesized to be responsible for selecting and triggering articulatory motor programs, while suppressing others, at the appropriate time during speech sequencing.

There is an extensive body of evidence (recently reviewed by Chang & Guenther, 2020) in favor of the idea that stuttering involves an inability to initiate, sustain, and/or terminate speech motor programs due to anomalous basal ganglia function. For example, numerous acoustic investigations have provided evidence that stuttering involves an impaired ability to transition from the articulatory gestures affiliated with an initial phoneme of a syllable to subsequent gestures, as indicated by protracted voice onset times and formant transition rates (e.g., Boutsen, 1993; Chang et al., 2002; Robb & Blomgren, 1997; Robb et al., 1998; Yaruss & Conture, 1993). As for the underlying neural circuitry, abnormal functional activity in the basal ganglia has frequently been implicated in stuttering severity (Connally et al., 2018; Giraud et al., 2008; Ingham et al., 2012; Toyomura et al., 2015). In addition, stuttering-like disfluencies have been reported to emerge in neurodegenerative diseases that impair the function of the basal ganglia, such as Parkinson’s disease (Niethammer et al., 2012), and deep brain stimulation applied to either the subthalamic nucleus or the globus pallidus pars interna of the basal ganglia has been shown to relieve or exacerbate stuttering disfluencies (see Sapir, 2014, and Skodda, 2012, for reviews).

Moreover, and of particular relevance to the current research, some existing theoretical models attribute a role in speech motor sequence learning to the cortico-BG loops (e.g., Guenther, 2016, Chapters 8 & 10; Kotz & Schwartze, 2010; see also Fee & Goldberg, 2011, for a similar account in songbird vocal learning). For example, in the Gradient Order Directions Into Velocities of the Articulators (GODIVA) model of speech sequencing and initiation (Bohland et al., 2010; Civier et al., 2013; Guenther, 2016, Chapter 8; Segawa, Masapollo, et al., 2019), it is proposed that continual speech motor practice leads to the formation of optimized motor programs for frequently occurring kinematic sequences, such as the successive gestures making up a word or syllable (see also, Cholin et al. 2006). These learned movement “chunks” or “templates” are then encoded and sequentially activated (or “read out”) by the cortico-BG loops during production. The fundamental idea is that the cortico-BG loops perform two distinct, but related, cognitive operations during the speech production process, namely, forming regularized motor chunks and initiating/activating motor programs affiliated with those chunks.

Results from a functional magnetic resonance imaging (fMRI) study of speech motor sequence learning with neurotypical adults (Segawa, Tourville, et al., 2015) provided empirical support for this view. In the initial training phase of the study, participants performed a cued motor sequence learning task (outside the scanner) in which they intensively practiced producing pseudowords (monosyllabic CCVCC[C], where C = consonant, V = vowel) consisting of consonant clusters that were either phonotactically legal in their native language of English (e.g., “BLERK”) or illegal in English but legal in other natural human languages (e.g., “GVAZF”). The results indicated that, behaviorally, motor practice led to measurable performance gains (i.e., reduced error rates and shorter utterance durations), presumably because the sequences of independent speech movements had become concatenated into larger cohesive chunks (see Segawa, Masapollo, et al., 2019, for supporting evidence).

In the subsequent test phase (inside the scanner), the authors measured the blood oxygen-level dependent (BOLD) response during production of the practiced native and practiced non-native words as well as a novel set of non-native words (matched in phonetic complexity) using sparse sampling fMRI (see, e.g., Gracco et al., 2005). In one set of imaging analyses, BOLD activity associated with production of the novel non-native words was contrasted with that of the practiced non-native words. The results demonstrated increased activation in (left-lateralized) cortical (i.e., pre-supplementary motor area [preSMA], anterior insula [aINS], inferior frontal sulcus [IFS], inferior parietal sulcus [IPS], ventral premotor cortex [vPMC]), and subcortical basal ganglia (i.e., globus pallidus [GP]) regions involved in the cortico-BG loops (Alexander et al., 1986). It was further demonstrated that the degree of motor performance improvements between training and test was correlated positively with activity in the left aINS. The cortical regions that were found to be recruited in this task overlap with activation foci that have been identified in meta-analyses of working memory neuroimaging studies (see, e.g., Rottschy et al., 2012). Thus, these findings are in keeping with the hypothesis that the cortico-BG loops play an important role in learning and sequencing speech movement chunks since a higher number of chunks will have to be concatenated in a working memory repository (or phonological output store) and then subsequently read out during the production of novel sequences compared to practiced ones.

In another set of analyses contrasting BOLD activity associated with production of the practiced non-native and practiced native words, the authors found increased activation in a similar network of brain areas, although notably no increased activation was found in the left GP for this contrast. They did, however, report increased activation in the right cerebellum (lobule VI) for this contrast, suggesting that other subcortical structures that extend beyond the cortico-BG may also play a role in speech motor sequencing learning and motor control (a point we will return to in the general discussion).

Nevertheless, if the cortico-BG loops are involved in successively encoding and activating speech motor chunks during sequence learning and production, then that raises the possibility that the neural deficits hypothesized to underlie stuttering are related not only to problems with *motor execution*, but also to impaired *motor learning* mechanisms. Consistent with this view are experimental findings, in the behavioral literature, that adults who stutter (AWS) and adults with normal speech (ANS) often show differences in their ability to learn a variety of novel speech and nonspeech (i.e., finger tapping) movement sequences (e.g., Ludlow et al., 1997; Namasivayam & van Lieshout, 2008; Smits-Bandstra et al., 2006). Qualitatively similar findings have also been found in patients with Parkinson’s disease (Ferraro et al., 1993; Helmuth et al., 2000; Jackson et al., 1995; Mollaei et al., 2013; Smits-Bandstra & Gracco, 2013; Vakil et al., 2000).

### The Current Study

In the current research, we performed a functional brain-imaging study to distinguish between motor execution and motor learning impairments in AWS at both the behavioral and neural levels, by replicating and extending Segawa, Tourville, et al. (2015) with a cohort of adult speakers with persistent developmental stuttering. Toward this end, we trained AWS and ANS to produce pseudowords containing non-native consonant clusters. Participants were first trained over several days (outside the scanner) to produce two sets of novel CCVCC pseudowords: (1) syllables that involved native consonant clusters (*practiced native*) and (2) syllables consisting of non-native consonant clusters that are phonotactically illegal in English (*practiced non-native*). Based on previous results (Segawa, Tourville, et al., 2015; see also Segawa, Masapollo, et al., 2019), we expected to observe significantly larger performance gains throughout the course of training for the *practiced non-native* words than for the *practiced native* words (for which performance is already expected to be near ceiling at the beginning of training). During a subsequent testing phase (inside the scanner), we then contrasted BOLD activity associated with the production of the *practiced non-native* and *practiced native* words, and the *practiced non-native* and *practiced native* sequences.

We hypothesized that, if motor sequence learning mechanisms per se are impaired in stuttering, then: (1) At the behavioral level, AWS should show a slower rate of learning over the course of training and/or poorer learning outcomes compared to ANS; and (2) At the neural level, contrasting the BOLD signal for the *novel non-native*–*practiced non-native* and/or *practiced non-native*–*practiced native* conditions should yield less activity in the regions of the cortico-BG circuit previously identified in Segawa, Tourville, et al. (2015) in AWS, especially the left aINS, since they will not have fully formed cohesive motor chunks for the trained speech sequences.

If, however, the only core deficit underlying stuttering is related to motor implementation rather than to motor learning mechanisms, then: (1) At the behavioral level, AWS should show comparable performance gains over time but will display poorer (and/or slower) overall motor performance compared to ANS; and (2) At the neural level, AWS should show a similar reduction in activations in the aforementioned regions of the cortico-BG circuit implicated in motor sequences learning and working memory (the left aINS, preSMA, and IFS) as ANS, but should still show differences in primary motor (and possibly premotor) cortex; and (3) The size of the BOLD activity difference in (at least some of) these brain areas will covary with stuttering severity (and/or in-scanner disfluency) since mechanisms involved in selecting and initiating successive speech motor programs will be more impaired in AWS with more severe stuttering.

## MATERIALS AND METHODS

### Participants

Sixteen AWS (14 male, age range 18–42, median age 26) and fifteen ANS (13 male, age range 18–40, median age 25) were paid for participating in three testing sessions on three separate days. Participants reported normal (or corrected-to-normal) vision and no history of hearing, speech, language, or neurological deficits (apart from stuttering in the AWS group). All were right-handed (Oldfield, 1971; mean score = 80.46) and native speakers of American English with no previous experience with any of the languages used in stimulus creation (see following text). Individuals were excluded from taking part in the study if they were currently on medications that may have substantial effects on neural activity, or if they had claustrophobia preventing them from completing the MRI protocol. All participants underwent a magnetic resonance safety screening.

An experimenter interviewed all participants to confirm the diagnosis of persistent developmental stuttering in AWS and to confirm normal speech production in ANS. None of the AWS were enrolled in a fluency-shaping program at the time of participation. The stuttering severity of each AWS was assessed using the Stuttering Severity Instrument, Edition 4 (SSI-4; Riley, 2009). As part of this assessment, each AWS was video recorded while reading aloud, conversing with an experimenter, and speaking on the telephone. A certified speech-language pathologist then rated the frequency and duration of the stuttering events and the presence of physical concomitants that accompanied the moments of disfluency (e.g., eye-blinking). Stuttering severity in the AWS group ranged from 13 to 48, with a median of 27, and an interquartile range of 17 to 34. Five of the 16 AWS were categorized as “very mild” (SSI-4 score <17), 1 as “mild” (18–24), 5 as “moderate” (25–31), 3 as “severe” (32–36), and 2 as “very severe” (37–46).

### Stimuli

The speech stimuli consisted of several sets of monosyllabic pseudoword sequences (15 words per set). As shown in Table 1, all items contained either native or non-native syllable-initial (onset) and syllable-final (coda) consonant clusters. In the native sequences (e.g., “BLERK,” “THRIMF,” “TRALP”), the onset and coda clusters are phonotactically legal in English; in the non-native sequences (e.g., “FPESCH,” “GVAZF,” “TPIPF”), the clusters are phonotactically illegal in English, but do occur in some other natural human language. The non-native clusters were taken from a variety of languages and language families including Hebrew, Leti, Taba, Romani, Polish, Lithuanian, Romanian, Georgian, Tepehua, Hungarian, and Pima; participants reported no prior experience with any languages in which these consonant clusters readily occur. None of the stimuli were an orthographic or a phonological word according to the MRC Psycholinguistic Database (Coltheart, 1981). All items containing non-native clusters had a neighborhood size of 0, and no real English words could be created by adding, deleting, or substituting a single phoneme in any subsequence. All of the clusters—both native and non-native alike—were bi- or triconsonantal and used in either onset position or coda position, but not both. Each cluster was used in only one word (i.e., no two words contained the same consonant cluster). The number of phonemes per word was counterbalanced across experimental conditions (see below).

**Table 1.** International phonetic alphabet (IPA) transcription and orthography for experimental stimuli used to elicit the *native* (left) and *non-native* (right) target onset and coda clusters

Phonotactics			
Native		Non-native	
IPA	Orthography	IPA	Orthography
blɚɹk	BLERK	fsɛfk	FSEFK
bɹalk	BRALK	fʃIkp	FSHIKP
dɹalf	DRALF	fθæmtʃ	FTHAMCH
flIsk	FLISK	fzItʃb	FZICHB
fɹʌmp	FREMP	vsɛpʃ	VSEPSH
glæntʃ	GLANCH	vðæʃp	VTHASHP
gɹalv	GRALVE	zvɛktʃ	ZVEKCH
klɛlθ	KLELTH	fpɛstʃ	FPESCH
kɹeInθ	KRENGTH	ftɛbstʃ	FTEBSCH
plaɹθ	PLARTH	ʃkɛvt	SHKEVT
pɹʌndʒ	PRENGE	ʃtæzg	SHTAZG
ʃɹidθ	SHRIDTH	vbImk	VBIMK
tɹælp	TRALP	vgæmʃ	VGAMSH
θɹImf	THRIMF	zbæpk	ZBAPK
dwIlm	DWILM	zdɛbg	ZDEBG
kwanst	KWANST	bvImpf	BVIMPF
gwɛfθ	GWEFTH	bzInstʃ	BZINSCH
twɚɹv	TWERVE	gvæzf	GVAZF
θwIlb	THWILB	kvætʃk	KVACHK
splɚɹst	SPLERST	tfIpʃtʃ	TFIPSHCH
spɹIdθ	SPRIDTH	tvItp	TVITP
swarf	SWARF	bdeŋt	BDANGT
skɛln	SKELN	dkɛdv	DKEDV
stIsp	STISP	gbɛsb	GBESB
		kpɛʃtʃ	KPESHCH
		ptætʃst	PTACHST
		tbæstf	TBASTF
		tgItk	TGITK
		tpIpf	TPIPF
		zgɛkf	ZGEKF

To create the prompts for the elicited production task, a female native speaker of American English was recorded producing the words. The model speaker was phonetically trained and had previously practiced producing the sequences until each stimulus could be executed fluently (i.e., without vocoid epenthesis or phoneme omissions, swaps, or substitutions). All recordings took place in a sound-attenuated booth. The speech was recorded directly to a computer using Audacity® software (Version 2.0.3, Audacity Team) via a microphone (Samson C01U studio condenser) connected to a pre-amplifier (44.1-kHz sampling rate, 32-bit quantization). The speaker recorded multiple randomized repetitions of each token. From these repetitions, one instance of each token was selected on the basis of clarity and acoustic similarity in voice pitch (*f0*) to the other stimuli in the set. Using Praat software (Boersma & Weenink, 2020), all recorded tokens were digitally edited to be matched for peak intensity and duration (i.e., 480 ms) without changing *f0*.

### Procedure and Design

Participants completed a cued sequence production task (see, e.g., Segawa, Masapollo, et al., 2019; Segawa, Tourville, et al., 2015). The experiment consisted of a training phase, during which participants learned to produce 15 words containing native clusters and 15 words containing non-native clusters, followed by a test phase, during which participants were tested on their ability to produce all 30 of the previously learned words and 15 novel words containing non-native consonant clusters. FMRI data were only collected during the test phase (see below). For both phases, participants were asked to repeat aloud each of the target words individually, which were presented both auditorily over noise-cancelling headphones and visually using text stimuli (as shown in Table 1). The training phase consisted of eight blocks of trials over two consecutive days (four on day one and four on day two). Each training block contained eight repetitions of each word, for a total of 240 trials per block. The test phase consisted of eight blocks of trials performed on day three (inside the MRI scanner) after completing the training phase (outside the scanner on days one and two). Each test block contained five to six repetitions of each word, for an average of 40 trials per block, from three stimulus categories (conditions): (1) *practiced native* words (i.e., words comprising native clusters that were previously encountered in the training phase), (2) *practiced non-native* words (i.e., words comprising non-native clusters that were previously encountered in the training phase), and (3) *novel non-native* words (i.e., novel words with novel clusters that were not encountered in the training phase).

Participants were divided into four groups, each of which practiced producing a different subset of the native and non-native sequences during training. The non-native words that were not practiced during training were used as novel non-native words during the imaging session. Assignment of non-native words to the *practiced non-native* and *novel non-native* categories was counterbalanced across participants. The training phase occurred one to two days before the test phase to allow for sleep-mediated memory consolidation of the newly acquired motor traces (see, e.g., Doyon, Albouy, et al., 2015; Doyon, Bellec, et al., 2009; Fenn et al., 2003; Vahdat et al., 2017; cf. Brawn et al., 2010; Pan & Rickard, 2015).

During training, participants were seated in a chair in front of a laptop (IBM Lenovo ThinkPad X61s) computer screen in a sound-treated laboratory room that was dimly lit. The auditory speech stimuli were presented over headphones (Sennheiser, HD 280 Pro) at a comfortable listening level, and utterances produced by the participants were recorded with a Samson (Hauppauge, NY) C01U USB studio condenser microphone connected to the computer via a MOTU microbook audio interface. Utterances were recorded using MATLAB (MathWorks Inc., Natick, MA) at 44.1 kHz. During testing, participants laid supine in an MRI scanner. Instructions and visual stimuli were projected onto a screen viewed from within the scanner via a mirror attached to the head coil. The auditory stimuli were played over Sensimetrics model S-14 MRI-compatible earphones. Participants’ productions were transduced by a Fibersound model FOM1-MR-30m fiber-optic microphone, sent to a Lenovo ThinkPad X61s, and recorded using MATLAB at 44.1 kHz.

The trial structure was identical during training and testing. First, the orthographic display of a given syllable was centrally presented in tandem with its corresponding auditory prompt. Participants heard each prompt only once on each trial. Then, after the offset of the auditory presentation, a tone was presented for 50 ms. The time between stimulus offset and tone onset was randomly jittered between 500 and 1,000 ms. This tone served as a “go” signal that prompted the participant to go ahead and repeat the token as clearly and accurately as possible. For all phases, participants produced the target syllables in a pseudo-random order. The combination of the auditory and orthographic presentations was necessary because prior studies have shown that listeners tend to perceive non-native consonant clusters as epenthesized disyllabic sequences (e.g., Berent et al., 2007; Davidson & Shaw, 2012; Dupoux, Kakehi, et al., 1999; Dupoux, Parlato, et al., 2011; Pitt, 1998). Moreover, in another study that directly examined the effects of stimulus input modality (audio only vs. audio and text) on speakers’ ability to produce non-native consonant clusters (Davidson, 2010), it was found that the presence of text led to an improvement in overall task performance.

For both phases, participants were instructed to repeat the target syllable as clearly and accurately as possible as soon as they heard the tone. Several familiarization trials with experimenter feedback were included at the start of the experiment to confirm that participants understood the task instructions and were able to perform the task. The sequences used during these initial practice trials were not used at any point in the rest of the study. Feedback was not provided during the actual training or test phases.

### Behavioral Data Analyses

We conducted several analyses to provide evidence, at the behavioral level, that incremental motor learning occurred throughout the two days of speech motor practice (outside the MRI scanner), and that such learning was retained at test (inside the scanner). In the first analysis, we analyzed three temporally sensitive measures of learning across the eight training blocks (on days 1 and 2): (1) articulatory sequencing error rate, (2) utterance duration (interval from utterance onset to offset), and (3) reaction time (RT) (interval from the offset of the go-signal to utterance onset). These measures are generally believed to quantify the ease or difficulty with which speakers retrieve and execute speech sequences (Sternberg et al., 1978) and are commonly used in both the motor sequence learning and second language learning literatures as measures of learning extent (see, e.g., Brawn et al., 2010; Buchwald et al., 2019; Doyon, Albouy, et al., 2015; Nakamura et al., 1998; Rauschecker et al., 2008; Segawa, Masapollo, et al., 2019). Sequencing errors were defined as phoneme additions (including schwa insertions), deletions, and substitutions, and utterance repetitions, omissions, and restarts. Based on prior findings reported with neurotypical speakers (Segawa, Masapollo, et al., 2019; Segawa, Tourville, et al., 2015), we expected to observe greater learning (at least in the ANS group) for the non-native sequences because those sequences included both novel words and novel onset and coda clusters, whereas the native sequences included novel words with familiar onset and coda clusters.

In the second analysis, to provide evidence of the *retention* of learning at test, we examined the same three performance measures for each experimental group (ANS vs. AWS) and condition (*practiced native* vs. *practiced non-native* vs. *novel non-native*) during the test phase (on day 3). For each word production, each measure was calculated following the removal of noise associated with the scanner bore echo and peripheral equipment using a Wiener filter (Wiener, 1949). The coder was blind to the condition (practiced vs. novel) of the non-native syllables. We hypothesized that if participants formed regularized motor chunks for the previously trained speech sequences, then they should perform more accurately (and/or faster) at generating the *practiced native* and *non-native* sequences compared to the *novel non-native* sequences.

In a final analysis, we examined whether, and if so, how, speech motor practice influenced fluency in the AWS group. Toward this end, we compared the mean number of trials perceptually coded as containing at least one stuttering-like disfluency during both the training and test phases (for the AWS group only) for each stimulus condition (*practiced native* vs. *practiced non-native* vs. *novel non-native*). Disfluencies were determined by a certified speech-language pathologist.

For each of these analyses, custom MATLAB software was used to perceptually rate and acoustically measure onsets and offsets of syllables by viewing the waveform and spectrogram and listening to the audio files. Listeners first marked, blinded to experimental condition, whether the target phoneme sequence was produced fluently. Disfluent productions were further categorized into four possible disfluency types: (1) repetition (i.e., a phoneme or syllable was repeated), (2) prolongation (i.e., a phoneme was prolonged), (3) audible or silent blocking (involuntary filled or unfilled pauses in speaking), and (4) clustered (i.e., more than one stutter type was produced for a given sequence).

Each utterance was then marked for four possible sequencing error subtypes: (1) approximation of the target (i.e., phoneme deletion/omission, insertion, substitution, vowel epenthesis); (2) unrecognizable from the target (i.e., an entirely different sequence was produced); (3) unfinished word (i.e., the sequence produced was not completed before the end of the recording window); and (4) silence (i.e., no sequence was produced at the time of recording). Since we were concerned with the learning of non-native phonotactics rather than subphonemic allophonic details, productions from either the model speaker or the participants were not judged on how natural they sounded in the language from which they were derived.

In addition, it is important to note that it was impossible to reliably distinguish involuntary “silent” pauses or blocks associated with stuttering from intentional speech onset delays with the audio-only recordings that we obtained in the scanner (although there were constraints on what the delay could be since the length of the recording window was fairly short). Thus, we suspect that trials with stuttering pauses were categorized as sequencing error subtype 4 (i.e., no sequence produced). This is a limitation inherent in the current study and all studies of this kind in stuttering (unless video recordings of articulatory behavior inside the scanner are obtained). In order to explore the possibility that some trials containing “silent” stuttering blocks were included in the neuroimaging analyses, we plotted histograms showing the distribution of RT scores for each stuttering speaker in the test phase (inside the scanner). Critically, these plots did not reveal a bimodal distribution with one peak having frequency values clustered to the right. This finding suggests that any potential blocks or pauses were likely categorized as silent errors (sequencing subtype 4 as described above) and were excluded from the imaging analyses, in which case, the BOLD responses would not have been contaminated by potential delays in RT. See the Supplementary Materials in the online supporting information located at https://www.mitpressjournals.org/doi/suppl/10.1162/nol_a_00027 for further details.

Mean disfluency rates for each subject were calculated as the percentage of trials that contained one or more disfluency error subtypes, and mean error rates for each subject were calculated as the percentage of trials that contained one or more error subtypes. For each production containing no sequencing or disfluency errors, utterance onset and offset were automatically labeled based on sound pressure level thresholds, then hand-checked. Note that, in the neuroimaging component of the study (described below), only trials in which participants produced the target sequence accurately and fluently were analyzed.

### FMRI Paradigm

In addition to the three speaking conditions (*practiced native* vs. *practiced non-native* vs. *novel non-native*), a silent baseline condition was intermixed during imaging in which participants viewed a series of asterisks on the screen instead of the orthographic stimulus and rested quietly instead of uttering a word. FMRI data were acquired using a sparse sampling protocol (see Belin et al., 1999; Gracco et al., 2005; Perrachione & Ghosh, 2013) that allowed participants to produce the target syllables during *silent* intervals between volume acquisitions. A single volume was acquired approximately 4 s after speech onset on each trial which aligns with the 4–6 s delay in peak BOLD response onset (Belin et al., 1999). By scanning between speech productions, this protocol avoids the influence of scanner noise on speaker performance and brain activity responses and image artifacts resulting from speech-induced motion of the head.

The cued sequence paradigm was identical to that used during training (outside of the scanner) except with an additional pause after the production of each syllable to temporally align the image acquisition to the expected peak of the hemodynamic response. As previously described, the test phase consisted of eight blocks of trials which corresponded to eight functional runs. A single volume was recorded on each trial and the delay between volumes was approximately 10 s. Each functional run contained five or six productions of each stimulus item, for a total of 40 trials per run (each run lasted approximately 6–7 min). This resulted in a total of 320 test trials (80 trials per condition, including baseline). Conditions were shuffled and pseudorandomly distributed across the eight functional runs with at least eight instances of each condition appearing in each run.

### Image Acquisition

All neuro-imaging data were acquired using a 3-Tesla Siemens TIM Trio scanner, equipped with a 32-channel head coil. T2*-weighted gradient echo-planar fMRI images were collected to assess BOLD responses during the test phase. Forty-one horizontal slices were collected in each functional volume (in-plane resolution = 3.1 mm^2^, slice thickness = 3 mm, gap = 25%, acquisition time = 2.5 s, echo time [TE] = 20 ms); volumes were automatically aligned to the anterior commissure–posterior commissure line. Prior to collecting functional data, a gradient-echo field map sequence was collected; the resulting magnitude and phase images enabled offline correction of magnetic field distortions in functional images during data preprocessing (see below; Jezzard & Balaban, 1995). Structural images were collected using a T1-weighted multi-echo MPRAGE pulse sequence (MEMPRAGE, voxel size = 1 mm^3^, 176 axial slices, 256 × 256 field of view, repetition time [TR] = 2,530 ms, TE = 3.44 ms, flip angle = 7°).

### FMRI Data Analysis

#### Image preprocessing

Functional data were processed using tools from the following software packages that were integrated into a processing stream within SPM12 (Statistical Parametric Mapping, v12; www.fil.ion.ucl.ac.uk/spm/): FreeSurfer (Dale et al., 1999; Fischl, Salat, et al., 2002; Fischl, Sereno, et al., 1999; www.freesurfer.net), Artifact Detection Tools (ART; www.nitrc.org/projects/artifact_detect/), and the CONN toolbox (Whitfield-Gabrieli & Nieto-Castañón, 2012). Freesurfer was used to remove non-brain components of the T1 structural volumes; segment the brain into gray matter, white matter, and cerebral spinal fluid components; generate a reconstruction of the cortical surfaces of each hemisphere; and identify cortical and subcortical regions of interest (ROIs) (see below). Functional data were preprocessed through two pipelines: a surface/vertex-based pipeline for analysis of cortical responses and a volume/voxel-based pipeline for analysis of subcortical basal ganglia and cerebellar responses. Prior to pre-processing, the first volume of each functional series was removed because it served only as a trigger for the initial experimental trial.

A surface/vertex-based analysis pipeline was used to assess BOLD response differences for each experimental group and for each contrast in the cerebral cortex. Functional images from each subject were simultaneously realigned to the mean subject image and unwarped (motion-by-inhomogeneity interactions) using the SPM12 realign and unwarp procedure (Andersson et al., 2001). Outlier scans were detected using ART based on framewise displacement (scan-to-scan motion threshold of 0.9 mm) and mean signal change (scan-to-scan signal change threshold of 5 standard deviations above the mean (see Nieto-Castañón, 2020, for details). Framewise displacement was computed at each timepoint by considering a 140 × 180 × 115 mm bounding box around the brain and estimating the largest displacement among six control points placed at the center of the bounding-box faces. Global BOLD signal change was computed at each timepoint as the change in average BOLD signal within SPM's global-mean mask scaled to standard deviation units. Functional volumes from each subject were then coregistered with their high-resolution T1 structural images and resliced using SPM12’s inter-modal registration procedure with a normalized mutual information objective function. The functional data were then resampled at the location of the FreeSurfer *fsaverage* level-8 tessellation (163,842 vertices and 327,680 faces) projected on each subject-specific cortical surface, averaged across 10 intervals along the normal between the white matter and pial surfaces, and smoothed using iterative diffusion smoothing with a series of 40 discrete steps, approximately equivalent to a 8 mm full-width half-maximum (FWHM) two-dimensional Gaussian smoothing kernel (Hagler et al., 2006; Nieto-Castañón, 2020).

A volume/voxel-based analysis pipeline was used to identify differences in subcortical BOLD responses. Following the realignment, unwarping steps described above, functional volumes, and the original T1 structural volumes, were simultaneously segmented and normalized directly to Montreal Neurological Institute (MNI) space using SPM12’s combined normalization and segmentation procedure (Ashburner & Friston, 2005). Prior to MNI-normalization, both the functional and anatomical volume origins were centered to coordinates [0, 0, 0] in order to improve the quality of the iterative procedure initial starting estimates. Functional volumes were then spatially smoothed using an 8 mm FWHM Gaussian kernel in order to increase BOLD signal-to-noise ratio and reduce the influence of residual variability in functional and gyral anatomy across subjects (Nieto-Castañón, 2020).

#### Subject-level BOLD contrast analyses

After preprocessing, BOLD responses were estimated using a general linear model in SPM12. Because functional volumes were acquired in a sparse sequence, the hemodynamic response function for each stimulus event was modeled as a finite impulse response. The model included four condition-specific variables (practiced native, practiced non-native, novel non-native, and baseline). Trials with productions that were perceptually rated as containing either a sequencing error (e.g., phoneme deletions, insertions or substitutions) or a stuttering-like disfluency (e.g., phoneme/syllable repetitions, prolongations, or blocks), or were found to be outliers by ART were modeled as separate conditions (one individual regressor per trial) thereby removing variability resulting from these trials from the effects- and contrast-of-interest estimates. For each individual run, regressors were added to the model to remove linear effects of time (e.g., signal drift, adaptation) and the six motion covariates (taken from the realignment step) and a constant term.

The model was estimated at each vertex (surface-based) analysis or voxel (volume-based) analysis for each participant, resulting in two surface maps (one for each hemisphere) and a volume map of the model regressor coefficients for each condition. These condition estimates were then contrasted to yield effect-size maps of the following contrasts of interest: differences in the response during *novel non-native* and *practiced non-native* conditions (*novel non-native*–*practiced non-native*), and differences in the response during the *practiced non-native* and *practiced native* conditions (*practiced non-native*–*practiced native*).

#### Group-level analyses

Group-level *t* statistics were calculated separately for each contrast map. Cluster-level inferences were based on Threshold Free Cluster Enhancement (TFCE; Smith & Nichols, 2009) with default parameters extent = 0.5 and height = 2. This method assigns TFCE scores to each voxel characterizing the amount of cluster-like local spatial support at each location. These scores are then compared to their expected distribution under the null hypothesis, estimated using 1,000 randomization/permutation iterations, and the results are thresholded at family-wise error (FWE) false positive probability (*p*_FWE_) < 0.025. This threshold was used as a conservative means to ensure a cluster-level *p*_FWE_ < 0.05 across the cortical surface and subcortical volume analyses.

### Region-of-Interest-Based Analyses

To increase statistical power and sensitivity, we supplemented the surface/vertex-based and volume/voxel-based analyses with ROI analyses (Nieto-Castañón et al., 2003) based on a priori hypotheses derived from the results of Segawa, Tourville, et al. (2015). Specifically, we used anatomically defined ROIs in the speech production network that overlapped areas of significant BOLD activity in Segawa, Tourville, et al. (2015) for each contrast of interest. The predefined cortical and subcortical (basal ganglia and cerebellar) ROIs are listed in Table 2; a detailed description of the anatomical landmarks used for cortical parcellation are provided in Tourville and Guenther (2003). For the *novel non-native*–*practiced non-native* contrast, we tested whether brain activity was significantly different in globus pallidus internal (GPi) and external (GPe) segment ROIs; these ROIs were derived from the probabilistic atlas of the basal ganglia described by Keuken et al. (2014). For the *practiced non-native*–*practiced native* contrast, we tested whether brain activity was significantly different in right cerebellum lobule VI, which was derived from the SUIT probabilistic atlas of the cerebellum (Diedrichsen et al., 2009). Affirmation of differences in these ROIs would replicate the Segawa, Tourville, et al. (2015) findings with neurotypical speakers.

**Table 2.** List of the predefined cortical, subcortical, and cerebellar regions-of-interest (ROIs) used in the current analyses

Novel non-native–practiced non-native contrast
*Subcortical ROIs*
L GPi
L GPe

Practiced non-native−practiced native contrast
*Subcortical ROIs*
R cerebellum lobule VI

Brain-behavior motor learning correlation analyses
*Cortical ROIs*
L aINS
R aINS
L PT
R preSMA
L vPMC
L pSTS
L midPMC
L aFO
R aFO
L pIFS
L STG
L STS
*Subcortical ROIs*
L GPi
L GPe

Brain-behavior stuttering severity correlation analyses (AWS only)
*Cortical ROIs*
L vPMC + midPMC + vMC + midMC
L preSMA + SMA
*Subcortical ROIs*
L GPi
L GPe
L putamen
L caudate
L VA
L VL

### Brain-Behavior Correlation Analyses

Two types of hypothesis-driven analyses were conducted to identify potential relationships between behavioral measures and brain activity. In the first type of analysis, we conducted multiple correlation tests to identify relationships between behavioral measures of motor sequence learning success and BOLD activity. Specifically, we tested for correlations between (i) the mean BOLD response in the cortical and subcortical ROIs that showed task-activated clusters in Segawa, Tourville, et al. (2015; Table 2) in both the *novel non-native*–*practice non-native* and the *practiced non-native*–*practiced native* contrasts and (ii) each of the three motor learning indices (error rate, utterance duration, and RT). No correction was applied for the number of ROIs in this ROI list. FreeSurfer was used to define the ROIs on each individual cortical surface using the labeling system described in Cai et al. (2014). These ROIs included the left-lateralized aINS, vPMC, anterior frontal operculum (aFO), preSMA, IFS, posterior superior temporal sulcus (pSTS), and planum temporale (PT), as well as the left GPi and GPe. For each participant, we computed each motor learning index by subtracting the mean performance difference between the *novel non-native* and *practiced non-native* productions. We normalized for differences between participants by dividing these differences by the *practiced non-native* syllable measure, on a per-participant basis. For example, the utterance duration learning measure was the mean duration difference between the *novel non-native* and *practiced non-native* productions divided by mean duration of the *practiced non-native* productions. Each motor learning index was then correlated with the mean beta coefficient within each significant cluster from each contrast.

In the second type of analysis, SSI measures (Riley, 2009) of stuttering frequency and overall severity (described above) were used, in addition to the number of disfluencies produced during the test phase (inside the scanner), to identify potential relations between stuttering-related behaviors and brain activity. Specifically, we tested for correlations in the AWS group only with the mean composite SSI scores and mean disfluency rates during test (inside the scanner) and the mean BOLD responses in eight hypothesis-based ROIs within the cortico-BG loops (listed in Table 2) in the *novel non-native*–*practice non-native* contrast (based on fluent trials only). These ROIs included left premotor and primary motor areas (ventral and mid premotor cortices [vPMC, midPMC] + ventral and mid motor cortices [vMC, midMC]), left medial prefrontal areas (left SMA + preSMA), as well as several left-lateralized subcortical basal ganglia sites (GPi, GPe, caudate, putamen, ventral anterior nucleus [VA], ventral lateral nucleus [VL]).

## RESULTS

### Behavioral Measures of Speech Motor Sequence Learning

#### Training phase

**Figure 1.**
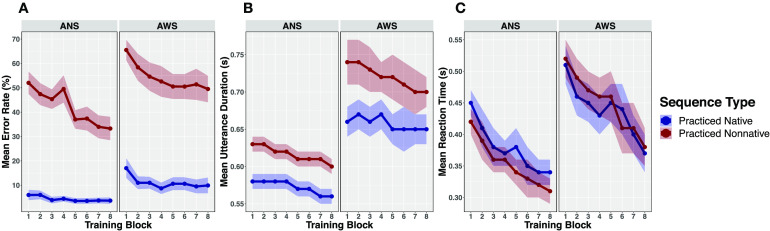
Time course of improvement of behavioral performance measures during training (outside the scanner) for the *practiced native* and *practiced non-native* sequences as a function of training block (1–8) and group (ANS vs. AWS). (A) Mean percentage of errors of each sequence type; (B) Mean durations of the properly executed utterances of each sequence type; (C) Mean reaction times of the properly executed utterances of each sequence type. The shaded area represents the standard error of the mean.

Table 3 shows a summary of the behavioral results for each phase of the experiment and for each group (ANS vs. AWS). Our first set of analyses examined, at the behavioral level, whether participants showed evidence of incremental speech motor sequence learning over the course of the training phase by examining the time course of improvement in each of the three performance measures—mean error rates, utterance durations, and RTs—over the two days of speech motor practice. Each performance measure was averaged within each group, training block, condition, and participant. Again, duration and RT measures were only analyzed for utterances that were perceptually coded as having been properly executed (see above) on each day. To visualize the running estimate of the learning trajectories across participants, Figure 1 plots the time course of improvement of each performance measure as a function of group, training block, and condition. Separate analyses of variance (ANOVAs) were performed on the mean sequencing error rates, durations, and RTs with training block (1–8) and condition (native vs. non-native) as within-subjects factors and group (ANS vs. AWS) as a between-subjects factor. In these and all subsequent ANOVAs, Greenhouse-Geisser corrections were applied when appropriate and partial eta-squared effect sizes were calculated for all main effects and interactions. Post-hoc pairwise comparisons were reported as significant at the 0.05 level.

**Table 3.** ANOVA results for behavioral analyses

Training phase					Test phase				
Dependent measure: Error rates									
Effect	*F*	*df*	*p*	*η*_*p*_^2^	Effect	*F*	*df*	*p*	*η*_*p*_^2^
Block	15.985	7	<0.001	0.355	Block	5.291	7	<0.001	0.159
Condition	208.822	1	<0.001	0.878	Condition	91.164	2	<0.001	0.765
Group	5.036	1	0.033	0.148	Group	1.371	1	0.251	0.047
Block × Condition	8.863	7	<0.001	0.234	Block × Condition	1.504	14	0.106	0.051
Block × Group	1.554	7	0.183	0.051	Block × Group	1.164	7	0.325	0.04
Condition × Group	1.137	1	0.295	0.038	Condition × Group	0.838	2	0.438	0.029
Block × Condition × Group	1.312	7	0.268	0.043	Block × Condition × Group	0.647	14	0.825	0.023

Dependent measure: Utterance duration									
Effect	*F*	*df*	*p*	*η*_*p*_^2^	Effect	*F*	*df*	*p*	*η*_*p*_^2^
Block	2.278	7	0.09	0.073	Block	2.638	7	0.033	0.083
Condition	34.318	1	<0.001	0.542	Condition	40.733	2	<0.001	0.584
Group	8.963	1	0.006	0.236	Group	0.112	1	0.74	0.004
Block × Condition	2.066	7	0.049	0.066	Block × Condition	1.118	14	0.354	0.037
Block × Group	0.132	7	0.931	0.005	Block × Group	0.517	7	0.738	0.018
Condition × Group	0.506	1	0.483	0.017	Condition × Group	0.949	2	0.357	0.032
Block × Condition × Group	1.358	7	0.25	0.045	Block × Condition × Group	1.059	14	0.388	0.035

Dependent measure: Reaction time									
Effect	*F*	*df*	*p*	*η*_*p*_^2^	Effect	*F*	*df*	*p*	*η*_*p*_^2^
Block	16.742	7	<0.001	0.366	Block	3.114	7	0.039	0.097
Condition	0.487	1	0.491	0.017	Condition	1.688	2	0.2	0.054
Group	1.934	1	0.175	0.063	Group	5.926	1	0.021	0.17
Block × Condition	1.938	7	0.094	0.063	Block × Condition	1.012	14	0.376	0.034
Block × Group	0.36	7	0.807	0.012	Block × Group	0.851	7	0.455	0.029
Condition × Group	3.732	1	0.063	0.114	Condition × Group	4.973	2	0.012	0.146
Block × Condition × Group	1.099	7	0.363	0.037	Block × Condition × Group	0.805	14	0.664	0.027

Dependent measure: Disfluency rates (AWS only)									
Effect	*F*	*df*	*p*	*η*_*p*_^2^	Effect	*F*	*df*	*p*	*η*_*p*_^2^
Block	2.618	7	0.084	0.158	Block	1.095	7	0.372	0.068
Condition	1.379	2	0.26	0.09	Condition	9.959	2	0.001	0.399
Block × Condition	2.038	14	0.108	0.127	Block × Condition	0.923	14	0.482	0.058

*Note*. Shown are the *F* value, the degrees of freedom, *p* value, and partial-eta-squared value for each effect.

The ANOVA performed on mean error rates (shown in Figure 1A) revealed a significant main effect of group [*F*(1, 29) = 5.036, *p* = 0.033, *η*_*p*_^2^ = 0.148], such that AWS [mean (*M*) = 32.7, standard deviation (*SD*) = 13.6] produced more sequencing errors than ANS [*M* = 23.0, *SD* = 10.3] in general. There were also highly significant main effects of condition [*F*(1, 29) = 208.822, *p* < 0.001, *η*_*p*_^2^ = 0.878] and block [*F*(7, 203) = 15.985, *p* < 0.001, *η*_*p*_^2^ = 0.355]. There was one significant interaction, the stimulus condition × block interaction [*F*(7, 203) = 8.863, *p* < 0.001, *η*_*p*_^2^ = 0.234]. All other interactions were not significant [*p* > 0.05, in all cases]. Simple effects tests on the condition × block interaction revealed that there was a significant effect of block on both the native sequences [*F*(7, 210) = 2.958, *p* = 0.045, *η*_*p*_^2^ = 0.090] and the non-native sequences [*F*(7, 210) = 20.495, *p* < 0.001, *η*_*p*_^2^ = 0.406]. However, the effect size was considerably larger in the non-native condition, indicating that sequence learning was greater for novel words with unfamiliar consonant clusters than novel words with familiar consonant clusters during training. This is in keeping with the behavioral results reported by Segawa and colleagues (Segawa, Tourville, et al., 2015; Segawa, Masapollo, et al., 2019) which found that relatively less learning occurred for native compared to non-native sequences.

The ANOVA performed on mean utterance duration (shown in Figure 1B) revealed a main effect of group [*F*(1, 29) = 8.963, *p* = 0.006, *η*_*p*_^2^ = 0.236], such that ANS produced shorter utterances [*M* = 0.59, *SD* = 0.05] than AWS [*M* = 0.69, *SD* = 0.10]. There was also a significant effect of stimulus condition [*F*(1, 7) = 34.318, *p* < 0.001, *η*_*p*_^2^ = 0.542], such that the native sequences [*M* = 0.61, *SD* = 0.09] were uttered faster than the non-native sequences [*M* = 0.67, *SD* = 0.10], as well as a condition × block interaction [*F*(7, 203) = 2.066, *p* = 0.049, *η*_*p*_^2^ = 0.066]. All other interactions were not significant [*p* > 0.05, in all cases]. Consistent with our expectations based on our prior studies, simple effects tests on the condition × block interaction revealed that there was a significant effect of block on the non-native sequences [*F*(7, 210) = 3.340, *p* = 0.002, *η*_*p*_^2^ = 0.100] but not on the native sequences [*F*(7, 210) = 1.038, *p* = 0.382, *η*_*p*_^2^ = 0.033]. Thus, participants only got reliably faster at executing novel words during training if they contained unfamiliar consonant clusters, likely because they were near ceiling performance for novel words using native clusters.

The ANOVA performed on mean RT scores (shown in Figure 1C) revealed a highly significant effect for block [*F*(7, 203) = 16.742, *p* < 0.001, *η*_*p*_^2^ = 0.366], such that participants got faster at initiating their utterances during the training sessions. The effects of group, condition, and all other interactions were not significant [*p* > 0.05 in all cases].

**Figure 2.**
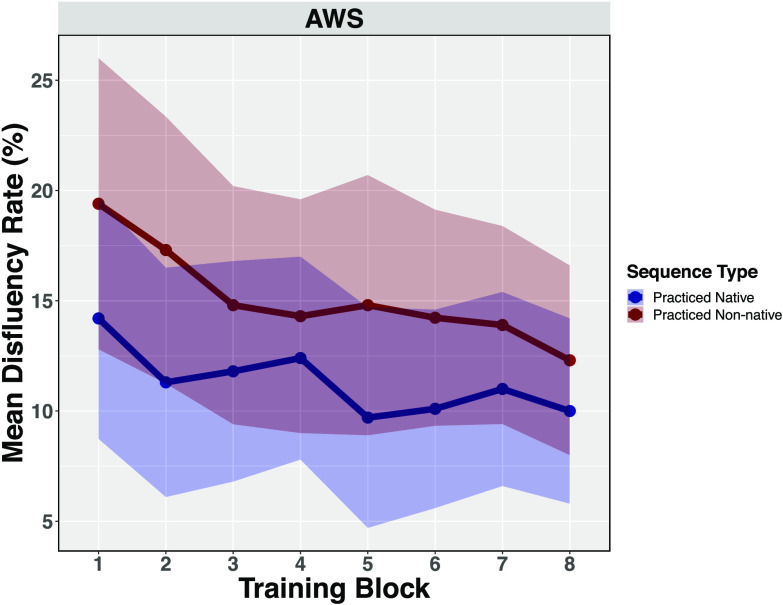
Mean percentage of disfluency errors for the *practiced native* and *practiced non-native* sequences during training (outside the scanner) as a function of training block (1–8) for AWS only. The shaded area represents the standard error of the mean.

In an additional analysis, we examined the mean disfluency rates for the AWS group only. These are shown in Figure 2 averaged across each sequence type (native vs. non-native) and block (1–8). An ANOVA on these scores—sequence type × block—showed no significant main effects or interaction [*p* > 0.05 in all cases], indicating that stuttering speakers were equally disfluent for both sequence types throughout training.

#### Test phase

**Figure 3.**
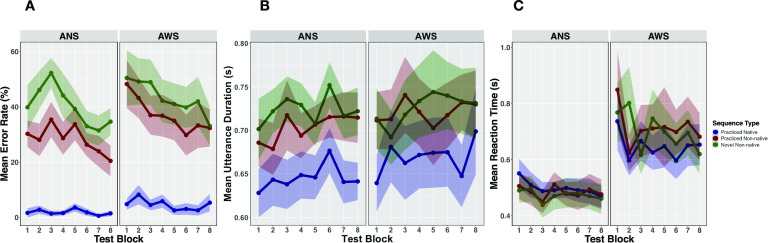
Behavioral performance measures during test (inside the scanner) for the *practiced native*, *practiced non-native*, and *novel non-native* sequences as a function of test block (1–8) and group (ANS vs. AWS). (A) Mean percentage of errors of each sequence type; (B) Mean durations of the properly executed utterances of each sequence type; (C) Mean reaction times of the properly executed utterances of each sequence type. The shaded area represents the standard error of the mean.

Our second set of analyses examined the same set of behavioral measures of sequence generation while participants were inside the MRI scanner during the test phase. Each performance measure was averaged within each group, testing block, condition, and participant. Duration and RT measures were again only analyzed for utterances coded as properly sequenced productions (see above) on each day. Table 4 shows the mean error rates during the test phase as a function of error subtype and sequence type. Figure 3 shows the mean error rates, utterance duration, and RT scores as a function of group, test block, and condition. Separate ANOVAs were performed on the mean error rates, utterance durations, and RTs with group (AWS vs. ANS) as a between-subjects factor, and test block (1–8) and experimental condition (*practiced native* vs. *practiced non-native* vs. *novel non-native*) as within-subjects factors.

**Table 4.** Mean error rates in the test blocks (inside the scanner) by error subtype and sequence type

**Sequencing errors**	ANS			AWS		
	Sequence type			Sequence type		
	Practiced native	Practiced non-native	Novel non-native	Practiced native	Practiced non-native	Novel non-native
Approximation of target	14.6	30.7	39.4	32.5	33.3	37.6
Unrecognizable from target	0.0	0.0	0.0	0.3	0.1	0.3
Unfinished utterance	0.0	0.0	0.0	0.2	2.8	2.3
Silence	0.0	0.0	0.0	0.6	1.8	2.7

ANOVA performed on mean error rates (shown in Figure 3A) revealed significant main effects of block [*F*(7, 196) = 5.291, *p* < 0.001, *η*^2^ = 0.159] and condition [*F*(2, 56) = 91.164, *p* < 0.001, *η*^2^ = 0.765]. There was no significant main effect of group [*F*(1, 28) = 1.371, *p* = 0.251, *η*^2^ = 0.092] or interaction effects [*p* > 0.05 in all cases]. Post-hoc *t*-test comparisons performed on the main effect of condition indicated that, regardless of group, the mean error rates for the *practiced native* syllables [*M* = 4.0. *SD* = 7.2] were significantly lower than the *practiced non-native* syllables [*M* = 34.8, *SD* = 20.2, *t*(30) = −9.357, *p* < 0.001, Cohen’s *d* = 2.03] and *novel non-native* syllables [*M* = 44.7, *SD* = 21.0, *t*(30) = −10.978, *p* < 0.001, *d* = 2.59], and the mean error rates for the *practiced non-native* syllables were lower than the *novel non-native* syllables [*t*(30) = −4.653, *p* < 0.001, *d* = 0.48].

Measures of mean utterance duration (shown in Figure 3B) patterned similarly. ANOVA performed on mean utterance durations indicated that there were also significant main effects of block [*F*(7, 203) = 2.638, *p* = 0.033, *η*^2^ = 0.083] and condition [*F*(2, 58) = 40.733, *p* < 0.001, *η*^2^ = 0.584]. Again, there was no significant main effect of group [*F*(1, 29) = 0.112, *p* = 0.740, *η*^2^ = 0.004] or interaction effects [*p* > 0.05 in all cases]. Post-hoc *t*-test comparisons performed on the main effect of condition indicated that, regardless of group, participants were faster at executing the *practiced native* syllables [*M* = 0.67, *SD* = 0.15] than the *practiced non-native* syllables [*M* = 0.74, *SD* = 0.16, *t*(30) = −6.797, *p* < 0.001, *d* = 0.45] and *novel non-native* syllables [*M* = 0.77, *SD* = 0.19, *t*(30) = −7.454, *p* < 0.001, *d* = 0.58]. In addition, participants were faster at executing the *practiced non-native* syllables than the *novel non-native* syllables [*t*(30) = −3.044, *p* = 0.005, *d* = 0.17].

ANOVA performed on mean RT scores (shown in Figure 3C) showed no main effect of condition [*F*(2, 58) = 1.688, *p* = 0.200, *η*_*p*_^2^ = 0.054]. There were, however, significant main effects of block [*F*(7, 203) = 3.114, *p* = 0.039, *η*_*p*_^2^ = 0.097] and group [*F*(1, 29) = 5.926, *p* = 0.021, *η*_*p*_^2^ = 0.170], such that AWS took longer to initiate their utterances [*M* = 0.71, *SD* = 0.36] than ANS [*M* = 0.48, *SD* = 0.16]. The condition × group interaction effect also reached statistical significance [*F*(2, 58) = 4.973, *p* = 0.012, *η*_*p*_^2^ = 0.146]. Simple effects tests on the condition × group interaction indicated that the effect of condition approached significance in AWS [*F*(2, 30) = 3.555, *p* = 0.053, *η*_*p*_^2^ = 0.369], but not in ANS [*F*(2, 28) = 0.540, *p* = 0.589, *η*_*p*_^2^ = 0.037]. All other interactions were not significant [*p* > 0.05 in all cases].

**Figure 4.**
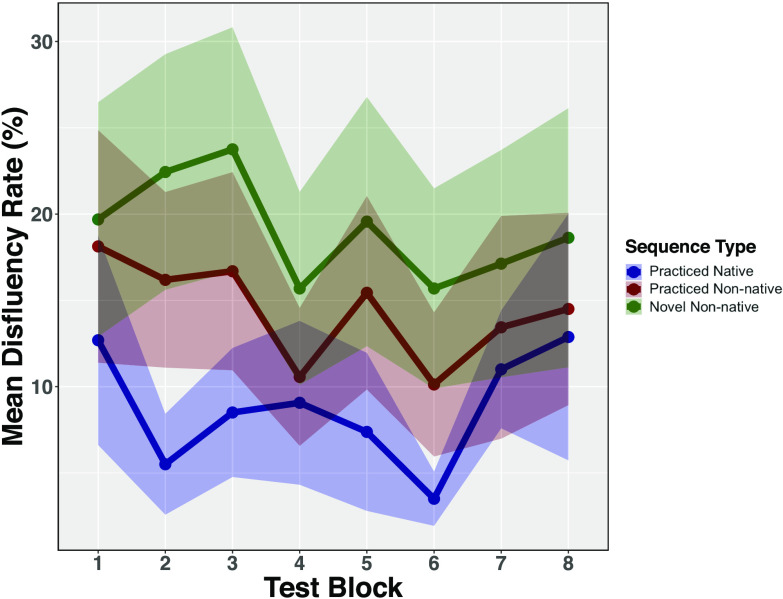
Mean percentage of disfluency errors (inside the scanner) for the *practiced native*, *practiced non-native*, and *novel non-native* sequences during test (inside the scanner) as a function of test block (1−8) for AWS only. The shaded area represents the standard error of the mean.

Finally, we again examined mean disfluency rates for the AWS group while in the scanner. These scores are shown in Figure 4. ANOVA performed on these scores—sequence type × block—revealed a highly significant main effect of condition [*F*(2, 30) = 9.959, *p* = < 0.001, *η*_*p*_^2^ = 0.399]. There was no significant main effect of block or interaction effect [*p* > 0.05 in both cases]. Post-hoc *t*-test comparisons performed on the main effect of condition indicated that AWS were less disfluent when executing the *practiced native* syllables [*M* = 4.6, *SD* = 11.8] compared to the *practiced non-native* syllables [*M* = 7.5, *SD* = 15.6, *t*(15) = −2.442, *p* = 0.027, *d* = 0.32] and *novel non-native* syllables [*M* = 9.8, *SD* = 19.4, *t*(15) = −3.476, *p* = 0.003, *d* = 0.49]. As well, AWS were less disfluent when producing the *practiced non-native* than the *novel non-native* syllables [*t*(15) = −2.307, *p* = 0.036, *d* = 0.19].

In summary, we obtained similar results to those in our prior studies on speech motor sequence learning with neurotypical speakers (Segawa, Tourville, et al., 2015; Segawa, Masapollo, et al., 2019): Participants—ANS and AWS alike—showed incremental improvements in performance speed and accuracy, especially for the non-native sequences, with repetition and practice during training and these gains were maintained overnight between experimental sessions. Moreover, the data indicated that, after two days of extended speech motor practice, speakers from both groups showed further performance gains in accuracy (reduced error rate) for the *practiced non-native* sequences throughout the test phase (Figure 3A). Although both groups showed comparable gains, the AWS produced the sequences slower and/or less accurately prior to and after training. Overall, these findings provide evidence at the behavioral level that AWS do not show deficits in the acquisition or retention of new speech motor sequences. Finally, RT scores were notably higher during test (inside the scanner) than during training (outside of the scanner), especially for the AWS group. Given that the experimental paradigm during the test phase was identical to that used during the training phase, this difference in initiation speed likely reflects differences in performance anxiety outside versus inside the scanner.

### Neural Correlates of Speech Motor Sequence Learning

#### FMRI analysis

**Figure 5.**
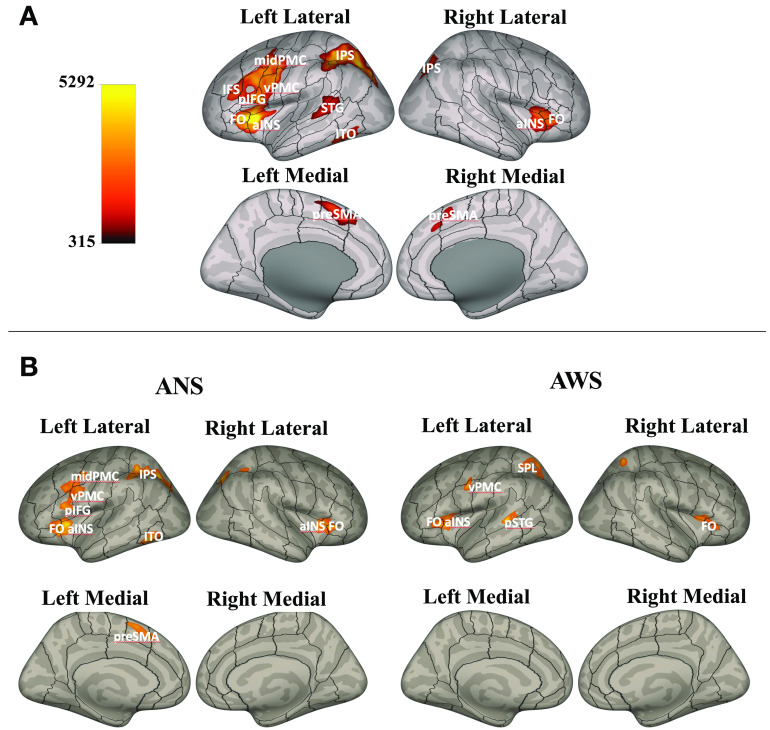
Brain areas showing greater BOLD activation for *novel non-native* than *practiced non-native* trials. (A) Averaged across all 15 ANS and 16 AWS; (B) 15 ANS (left) and 16 AWS (right). Activation is displayed on a canonical inflated cortical surface. Colors indicate the relative significance level at each voxel/vertex for the comparison of *novel non-native* and *practiced non-native* trials.

Before performing direct group comparisons, we first assessed differences between the condition-specific brain activations using the pooled results (ANS and AWS combined). For each of the two speech contrast conditions, we report the results from one-sided (positive only) tests. Figure 5A and Table 5 show the brain regions that were significantly more active during the execution of *novel non-native* than *practiced non-native* syllables (TFCE cluster-level *p*_FWE_ < 0.05) aggregated across both experimental groups. The surface/vertex-based analyses revealed that the production of *novel non-native* syllables resulted in greater BOLD response in preSMA, aINS, aFO, pFO, and IPS bilaterally. In the left hemisphere, additional cortical clusters were noted with peaks in IFS, vPMC, posterior inferior frontal gyrus (pIFG), midPMC, vMC, midMC, superior temporal gyrus (STG), and inferior temporal occipital (ITO) region. The volume/voxel-based analysis found no statistically significant differences in subcortical activity. We supplemented this voxel-based analysis with a hypothesis-based subcortical ROI analysis based on the results reported in Segawa, Tourville, et al. (2015) for this contrast. Specifically, we tested for differences in activation in the left GPi and GPe. Results from this analysis demonstrated that the left GPi was more active during production of the *novel non-native* than the *practiced non-native* words [*t*(30) = 2.22, *p*_*unc*_ = 0.034, *r* = 0.375], in effect replicating the finding of Segawa, Tourville, et al. (2015) for the same contrast. No significant groups effects (ANS vs. AWS) were found for any cortical or subcortical region in either hemisphere for the *novel non-native*–*practiced non-native* contrast; nevertheless, we present the results (albeit non-significant) for the two groups separately in Figure 5B.

**Table 5.** Summary of significant cortical and subcortical activation peaks for the *novel non-native*–*practiced non-native* contrast

Novel non-native–practiced non-native contrast*							
Anatomical region**	No. peaks	MNI coordinates of peak vertex			Size	TFCE	*p*_FWE_
		*x*	*y*	*z*			
Left hemisphere							
L pIFS, L aINS, L vPMC, L pFO, L midPMC, L aFO, L pMFG, L aIFS, L midMC, L dIFO, L vIFO, L IFR, L vMC, L aMFG, L FOC	33	−29	26	7	10170	5292.04	<0.0001
L SPL, L AG, L pSMG, L aSMG, L OC, L midSC	27	−27	−65	37	7665	4210.05	<0.0001
L ITO, L pITG, L TOF	7	−47	−59	−9	713	2009.43	0.001
L preSMA, L SFG, L SMA	3	−7	12	55	1150	1663.39	0.002
L pdSTS, L pSTG, L PT, L pvSTS	3	−63	−35	4	1662	1262.41	0.005
L pITG	5	−47	−43	−21	23	936.82	0.024

Right hemisphere							
R aINS, R pFO, R aFO, R IFR, R FOC	9	36	30	−1	2202	2562.18	<0.0001
R SPL, R AG, R OC	1	29	−62	35	1066	1923.23	0.002
R SFG, R preSMA	3	9	21	38	180	1003.95	0.019
R preSMA	1	7	16	55	267	953.0	0.024

*Note*. From left to right, the columns show the anatomical region name(s), number of peaks, MNI stereotactic coordinates, cluster size, TFCE value, and *p*_FWE_ value.

* For the one-sided (positive-only) contrast.

** Cluster regions are listed in descending order of number of significant vertices.

**Figure 6.**
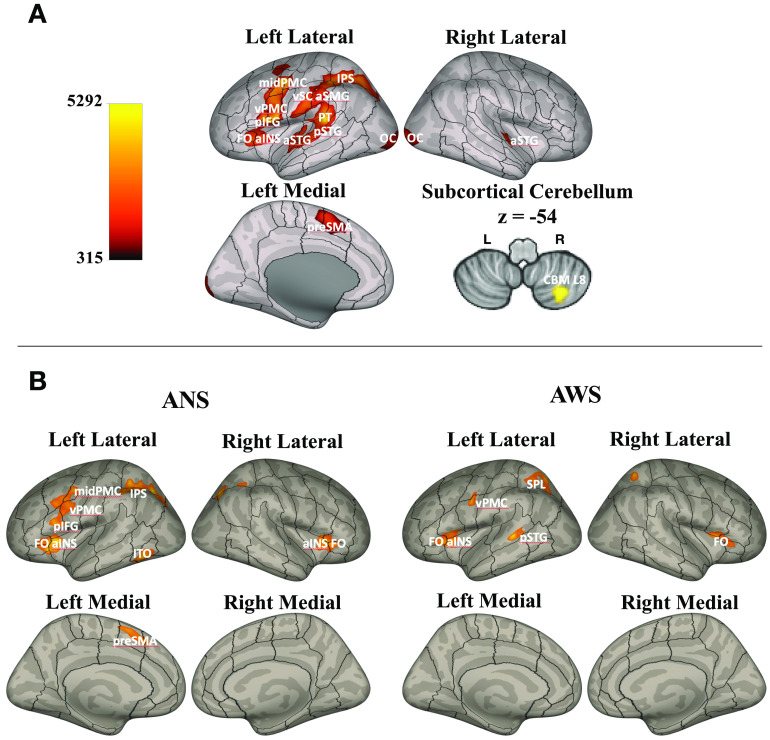
Brain areas showing greater BOLD activation for *practiced non-native* than *practiced native* trials. (A) Averaged across all 15 ANS and 16 AWS; (B) 15 ANS (left) and 16 AWS (right). Activation is displayed on a canonical inflated cortical surface. The significant subcortical cluster is shown on a slice through the cerebellum at *z* = −54 in the MNI template (panel A, bottom right); left and right hemispheres are indicated by L and R, respectively. Colors indicate the relative significance level at each voxel/vertex for the comparison of *novel non-native* and *practiced non-native* trials.

Figure 6A and Table 6 show the brain regions that were significantly more active during the execution of *practiced non-native* than *practiced native* syllables (TFCE cluster-level *p*_FWE_ < 0.05). The surface/vertex-based analyses revealed that the production of *practiced non-native* words resulted in greater BOLD response in pSTG and occipital cortex (OC) bilaterally. The increased activity in OC likely reflects greater attention and/or depth of processing while reading the non-native text stimuli, rather than differences in speech production. In the left hemisphere, additional cortical clusters were noted with peaks in preSMA, aINS, FO, pIFG, vPMC, midPMC, aSTG, ventral division of somatosensory cortex (vSC), and IPS. The volume/voxel-based analysis found increased activation in the right cerebellum (lobule XIII) during the *practiced non-native* than the *practiced native* condition. A hypothesis-based subcortical ROI analysis based on the results reported in Segawa, Tourville, et al. (2015) for this contrast was then performed. Specifically, we tested for differences in activation in right cerebellum (lobule VI). Results from this analysis demonstrated that this region was more active during production of the *practiced non-native* than the *practiced native* words [*t*(30) = 2.61, *p*_*FDR*_ = 0.014, *r* = 0.430], again replicating findings from Segawa, Tourville, et al. (2015). As in the other contrast, no region in either hemisphere was found to be significantly more active for this contrast in either group (ANS vs. AWS) at the cortical or subcortical levels, further bolstering the view that speech motor learning mechanisms are unimpaired in stuttering; again, we present the results (albeit non-significant) for the two groups separately in Figures 6B.

**Table 6.** Summary of significant cortical and subcortical activation peaks for the *practiced non-native*–*practiced native* syllable contrast

Practiced non-native–practiced native contrast*							
Anatomical region**	No. peaks	MNI coordinates of peak vertex			Size	TFCE	*p*_FWE_
		*x*	*y*	*z*			
Left hemisphere							
L SPL, L PT, L aSMG, L vSC, L PO, L pSTG, L H, L PP, L pSMG,L AG, L midSC, L pdSTS, L aSTG, L pCO, L OC, L adSTS, L pINS	47	−48	−38	13	13952	4603.4	<0.0001
L vPMC, L midPMC, L aINS, L pFO, L aFO, L vIFO, L vMC, L dIFO, L pIFS, L IFR, L aCO, L pMFG	17	−51	1	45	6766	3863.43	<0.0001
L preSMA, L SMA, L dCMA	1	−9	12	50	1246	1502.18	0.001
L OC	1	−15	−101	−7	2089	1246.82	0.009
L pMFG, L pdPMC, L mdPMC, L midPMC	1	−30	−8	46	726	977.68	0.019

Right hemisphere							
R PP, R H, R pINS	5	46	−18	2	407	1668.33	0.001
R OC	1	22	−100	6	1358	1583.67	0.001

Cerebellum lobule VIII	11	18	−70	−46	304	846.54	0.009
Cerebellum lobule VII b	1	32	−74	−56	1	645.83	0.024

*Note*. From left to right, the columns show the anatomical region name(s), number of peaks, MNI stereotactic coordinates, cluster size, TFCE value, and *p*_FWE_ value.

* For the one-sided (positive-only) contrast.

** Cluster regions are listed in descending order of number of significant vertices.

Although no significant groups effects were found, several interesting trends emerged that are worth noting and speculating about. First, brain areas linked to speech premotor planning (vPMC, midPMC, FO, aINS) showed a non-significant trend toward greater activation in ANS during novel sequence production, suggesting that neurotypical speakers may show greater learning of the motor chunks for the trained sequences than AWS. Second, STG, an auditory processing area, showed a non-significant trend toward greater activation in AWS during novel sequence production, suggesting that, following motor practice, stuttering speakers may still be relying more on auditory feedback to guide them toward the intended production targets compared to ANS. Critically, however, there were no brain areas activated in AWS that fell outside the network of areas identified in ANS and in the previous study (Segawa, Tourville, et al., 2015).

### Brain-Behavior Correlation Analysis

**Figure 7.**
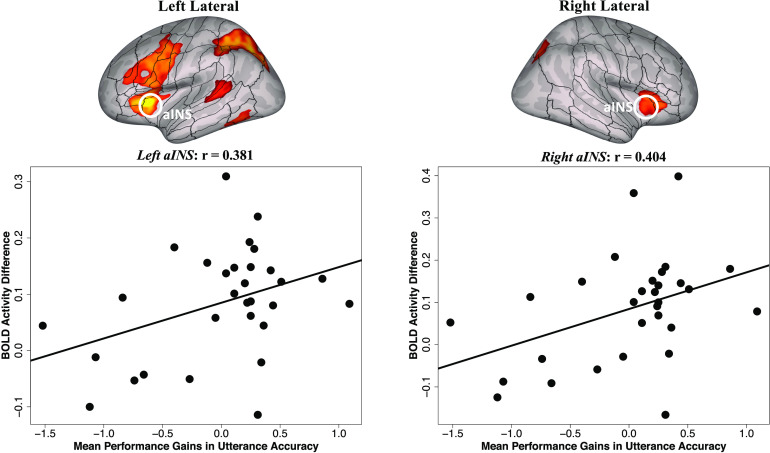
Significant correlation between participant-normalized difference in mean error rates between the *novel non-native* and *practiced non-native* words and the mean activation in the left aINS [*r* = 0.381, *p* = 0.034] (left panel) and right aINS [*r* = 0.404, *p* = 0.024] (right panel).

The correlation tests between behavioral measures of learning success and mean activation in the 12 hypothesis-based ROIs from Segawa, Tourville, et al. (2015) (listed in Table 2) for the *novel non-native*–*practiced non-native* contrast revealed no group differences at either the cortical or subcortical levels. In the analyses performed on the pooled data (ANS and AWS combined), we found that learning success, as measured by the participant-normalized difference in mean error rates between the *novel non-native* and *practiced non-native* words, was positively correlated with the mean activation in both the left [*t*(29) = 2.22, *p* = 0.034, *r* = 0.381] and right aINS [*t*(29) = 2.38, *p* = 0.024, *r* = 0.404], such that processing in these areas increased most with learning in participants who showed the greatest performance improvements (shown in Figure 7). These results provide support for the hypothesis that these premotor areas are involved in speech motor sequence learning. No significant correlations emerged between the participant-normalized differences in mean utterance duration (between the *novel non-native* and *practiced non-native* words) and the mean activation in any of the ROIs (in all cases, *p*_*unc*_ > 0.05).

**Figure 8.**
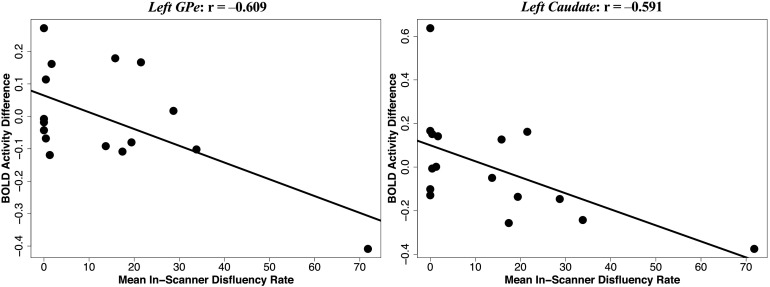
Significant correlation between mean disfluency rates during the test phase (inside the scanner) and mean activation in the left GPe [*r* = −0.609, *p*_*FDR*_ = 0.047] (left panel) and the left caudate [*r* = −0.591, *p*_*FDR*_ = 0.047] (right panel).

Finally, within the AWS group, we assessed whether stuttering severity (as indexed by SSI scores and mean disfluency rates inside the scanner during the test phase) were correlated with activation in eight hypothesis-based ROIs within the cortico-BG loops (listed in Table 2) for the *novel non-native*–*practiced non-native* contrast. Correlation tests revealed that mean disfluency rates inside the scanner were negatively correlated with mean activation in the left GPe [*t*(14) = −2.88, *p*_*FDR*_ = 0.047, *r* = −0.609] and the left caudate [*t*(14) = −2.74, *p*_*FDR*_ = 0.047, *r* = −0.591] (i.e., the more disfluent participants were during the test phase, the less BOLD activity change was observed in these basal ganglia sites for this contrast; shown in Figure 8). Although this result is correlational and therefore a causal relationship cannot be firmly established, it is nonetheless compatible with the idea that speakers are more likely to stutter when the left GPe and the left caudate are more hypoactive. In contrast, no statistically significant correlations were found between SSI scores and activation in any of the eight ROIs. The latter result is perhaps unsurprising given that the SSI does not measure disfluency during *non-native* sequence production and its affiliated scores were not based on speech uttered inside the scanner.

In summary, we found that, across both ANS and AWS, greater activity was observed during the production of novel sequences in brain regions previously associated with learning and maintaining speech motor programs, including lateral premotor cortex, FO, aINS, posterior superior temporal cortex, and right cerebellum (Guenther, 2016; Segawa, Tourville, et al., 2015). Measures of learning success correlated positively with activity in the left and right aINS, suggesting that this brain area plays an important role in speech motor learning. In AWS, measures of in-scanner disfluency rates were negatively correlated with activity in left-lateralized basal ganglia structures. Overall, these findings provide evidence that the neurobiological correlates of speech motor sequence learning do not differ across ANS and AWS.

## DISCUSSION

The goal of the current study was to investigate the behavioral and neural correlates of speech motor sequence learning in both stuttering and neurotypical adult speakers. Using a cued sequence production task, we examined changes in the performance of pseudowords containing non-native consonant clusters over time as a function of speech motor practice. At the behavioral level, we found that, although AWS were less accurate and/or slower compared to ANS at producing words involving non-native consonant clusters regardless of whether the words were practiced, they showed improvements in these measures with practice that were statistically comparable to those seen in ANS. At the neural level, we found that, across both groups and both speech contrasts (*novel non-native*–*practiced non-native* and *practiced non-native*–*practiced native*), practice producing words with non-native clusters led to significant decreases in BOLD activity in brain areas implicated in verbal working memory and speech motor planning (including the left preSMA, FO, aINS, and bilateral IPS), suggesting that processing load and articulatory effort decrease as the nervous system forms regularized motor programs (or chunks) for coordinated sequences of vocal tract gestures. Critically, there were no reliable group differences in either contrast map in any areas involved in the cortico-BG loops (or otherwise). Furthermore, the degree of motor performance gains in accuracy were correlated positively with activity in the left and right aINS. Collectively, these findings suggest that cortico-BG-mediated mechanisms involved in *learning* novel phoneme sequences are not impaired in AWS; instead, the impairment is primarily in the *motor execution* of speech sequences, novel and learned alike.

As in our prior study (Segawa, Tourville, et al., 2015), we also observed pre- to post-training BOLD activation changes in cortical areas that extend beyond those implicated in verbal working memory and speech motor planning processes. Specifically, we found greater activity in areas related to auditory processes (the left PT, aSTG, and pSTG) during production of the *novel non-native* sequences relative to the *practiced non-native* sequences (Figure 5). These auditory regions are thought to be involved in guiding speech movements based on self-generated auditory feedback (Guenther, 2016; Hickok, 2012). During speech production, activity in this area has been reported to be greater when there is a mismatch between predicted and actual auditory feedback (Tourville et al., 2008). Several existing theoretical models of speech production (e.g., Guenther, 2016; Hickok, 2012) propose that error signals arising from these regions are used to fine-tune speech motor programs over the course of repeated production attempts. Thus, learning is thought to rely on the transmission of these auditory error signals to frontal regions involved in motor planning and execution.

We also noted greater activity in cortical areas related to orthographic processing (the left ITO) during production of the *novel non-native* sequences relative to the *practiced non-native* sequences (Figure 5). Prior neuroimaging studies suggest that the ITO is a higher-level visual processing area involved in identifying letters and words from lower-level shape images (see, e.g., Pugh, Mencl, et al., 2001) and is therefore highly likely to be related to viewing and decoding the pseudowords that our participants were instructed to produce, rather than speech motor control. In contrast, production of the *practiced non-native* sequences did not produce significantly greater activity compared with *the practiced native* sequences in the ITO (Figure 6). This difference between the condition-specific brain activations suggests that pseudowords containing unfamiliar phonotactic sequences are more difficult to process prior to reading and repetition practice (cf. Pugh, Frost, et al., 2008).

The finding that the right cerebellum (which is structurally and functionally connected with left cortical areas) was recruited to a greater extent across both groups during the production of the *practiced non-native* than the *practiced native* sequences (shown in Figure 6) was also observed by Segawa, Tourville, et al. (2015) in ANS, and corroborates other reports with neurotypical participants that the cerebellum plays an important role in motor sequence learning and fine motor control (e.g., Ackermann, 2008; Doyon, Song, et al., 2002; Guenther, 2016, Chapter 2). For example, Bohland and Guenther (2006) found that different regions of the cerebellum showed differential sensitivity to syllable complexity (e.g., “stra” vs. “ta”) and serial complexity (e.g., “da-da-da” vs. “da-ru-ti”) in ANS, suggesting involvement in speech sequencing. That said, it is somewhat surprising that we did not find significant differences in cerebellar activity in the *novel non-native*–*practiced non-native* contrast. However, this comparison possessed less statistical power because of more removed error trials.

An alternative, but not mutually exclusive, interpretation to the cortical activity differences we found for both speech contrasts is that these differences result from the fact that the *novel non-native* and *practiced non-native* sequences are more difficult to *vocally imitate* than the *practiced native* sequences. Two sources of evidence support this conjecture. First, prior neuroimaging studies (e.g., Irwin et al., 2011) have reported that imitation of auditory speech produces additional significant activations in the left IFG and aINS when compared to a passive listening condition. Second, current computational models, such as the Directions Into Velocities of the Articulators (DIVA) model (Guenther, 2016), propose that speech motor learning is driven by initial mismatches between newly acquired sound targets and one’s own production attempts as represented in the auditory cortex. Auditory error signals are then transformed into corrective motor commands, and these corrective commands alter the feedforward commands for the next production attempt. As the feedforward commands improve, fewer error signals are generated and thus the contribution of the feedback control system gradually diminishes. The DIVA model thus predicts decreases in BOLD activation in both motor planning and auditory cortical areas as a consequence of imitation-based learning. In this view, the nervous system begins to form speech motor programs via an imitation-based learning mechanism, which may account for the increased activity in motor and auditory areas for the *novel non-native* compared with the *practiced non-native* sequences, and also for the *practiced non-native* compared with the *practiced native* sequences.

A number of other neuroimaging studies, compiled and discussed in a meta-analysis by Belyk et al., (2015), have reported that AWS tend to display higher activity in a number of right-hemisphere regions during fluently produced native speech when compared to ANS. Some researchers have proposed that this right-hemisphere cortical hyperactivity arises from impaired left-hemisphere function (see, e.g., Belyk et al., 2015; Fox et al., 1996; Guenther, 2016; Neef et al., 2017; cf. Connally et al., 2018). In view of these results, it is perhaps surprising that no clusters emerged in the right hemisphere for AWS in the current study. However, it is possible that right-hemisphere hyperactivity in AWS may not occur in all speaking situations. In particular, the current speaking task likely required more attention and articulatory effort than those in prior studies, since the participants were required to produce non-native sequences of segments, which is known to be difficult (e.g., Davidson, 2006, 2010). In addition, there may be methodological issues that concern the difference between the experimental procedures we used to test for a functional activation difference between ANS and AWS and the ones used by other research groups, as the existing literature on stuttering encompasses a diverse set of sampling protocols and motor tasks.

In the current study, it was further demonstrated that, in AWS, the in-scanner mean disfluency covaried with BOLD responses in left-lateralized basal ganglia sites (the GPe and left caudate), such that the more disfluent participants were during the test phase, the less BOLD activity change was observed in these basal ganglia sites for the *novel non-native*–*practiced non-native* contrast. This negative correlation does not establish a causal relationship; rather it establishes an associative link. Although this relationship is broadly consistent with the long-standing view that stuttering reflects a malfunction within the cortico-BG loops (e.g., Alm, 2004; Chang & Guenther, 2020; Connally et al., 2018; Fox et al., 1996; Giraud et al., 2008; Kell et al., 2009; Lu et al., 2010; Watkins et al., 2008), it would appear to be at odds with other neuroimaging studies (albeit that did not use a sparse-sampling method) which have reported *positive* relationships between basal ganglia activity and stuttering severity (e.g., Giraud et al., 2008; see also Metzger et al., 2018, for a similar finding based on manual motor responses in AWS). Differences between the findings of the current study and these prior studies suggest that the brain mechanisms underlying *general stuttering traits* and *transient disfluent states* are dissociable (for further discussion, see Connally et al., 2018).

At first glance, the lack of a robust learning difference between the ANS and AWS groups may appear to be at odds with other behavioral studies reporting motor learning deficits in AWS (Ludlow et al., 1997; Namasivayam & van Lieshout, 2008; Smits-Bandstra et al., 2006). Several important differences between those studies and ours might account for the divergent results. First, our study measured sequence and cluster learning using perception-based segmental transcription and simple acoustic measures. However, this approach required transcribers to make categorical decisions regarding the segments that speakers produced, and thus did not permit quantitative analysis of the presence or magnitude of various gestures, or of how speakers continually updated their implementation of novel gestural scores. In contrast, other motor learning studies (e.g., Namasivayam & van Lieshout, 2008) used kinematic measures to compare more subtle aspects of speech articulation between ANS and AWS. Thus, subtle differences in the articulatory correlates of speech motor learning may exist between ANS and AWS. Ongoing studies based on the current design that use electromagnetic articulography will determine whether AWS and ANS differ in their ability to learn to execute and coordinate inter-articulator movements for novel speech sequences.

Second, our study focused on the learning of new phoneme sequences within a single syllable with which the participants had no prior experience, since they violated English phonotactic constraints. This task required the learning of new articulatory sequences at a motoric level. In contrast, other studies in the literature on speech motor sequence learning in AWS have involved production of more extended, multi-syllabic sequences that conform to English phonotactics (e.g., Namasivayam & van Lieshout, 2008). However, there is evidence that multi-syllabic sequences place higher demands on phonological working memory mechanisms than on motor programming mechanisms. For example, in another fMRI study, McGettigan et al. (2011) found that activity in the PT, a region implicated in auditory working memory, increased as the number of syllables in either a non-word repetition or a passive listening task increased, whereas activity in the left SMA, a region implicated in speech motor programming, increased as the number of consonant clusters increased. In this light, the increased error rates reported in other studies with AWS might be interpreted instead as reflecting differences in phonological working memory mechanisms rather than motor learning or implementation mechanisms (see also Yang et al., 2019). Yet others have reported some differences between ANS and AWS in the degree of *motor adaptation* following an experimental perturbation to the online sensory feedback (e.g., Daliri et al., 2018), suggesting that mechanisms involved in *updating* existing motor programs may still be impaired in stuttering.

It is also important to note that the present fMRI measures focused on the *outcome* of speech motor sequence learning, not the *online process* of learning. However, previous research spanning a wide range of learning tasks and paradigms has shown that participants may have similar learning outcomes despite very different learning trajectories (see Karuza et al., 2014, for a review). Thus, despite showing comparable BOLD activity patterns at test, ANS and AWS may not show evidence of the same qualitative neural changes during the course of speech motor sequence training. It would therefore be of interest to conduct further fMRI studies, based on the current design, that perform BOLD contrast analyses throughout both the training and the test phases.

Finally, the current study focused primarily on the mechanisms of speech motor learning and control, from the phonetic encoding stage down to the motor commands to the speech articulators. Specifically, we examined how ANS and AWS learn to transform discrete phonological chunks (speech sounds that can be phonemes, syllables, or words) into a set of articulator movements that achieves the intended auditory “target.” We employed monosyllabic pseudoword stimuli to provide a “purer” measure of the speech motor system, as this would limit the recruitment of higher-level cognitive and linguistic processing strategies to assist task performance (see also McGettigan et al., 2011). However, any viable theory of speech production learning will ultimately have to explicate how these phonological chunks are integrated with prosodic structures, as well as their relation to syntactic/semantic planning processes. Future studies will aim to develop predictions from the highly controlled laboratory experiments presented here and elsewhere (e.g.,Segawa, Masapollo, et al., 2019; Segawa, Tourville, et al., 2015) that may be tested when speakers are instructed to produce novel speech sequences in a more natural context.

## ACKNOWLEDGMENTS

We are grateful to Barbara Holland and Diane Constantino for assistance with participant recruitment and/or data collection. We also thank Riccardo Falsini, Farwa Faheem, Abigail Cragin, Ariel Gordon, Angelise Bulit, and Jessica Smith for help with data analysis and visualization. Finally, this work benefited from helpful discussions with, or comments from, Elaine Kearney, Megan Thompson, Elizabeth Heller Murray, Jason Bohland, Cara Stepp, Kenneth Logan, and Tyler Perrachionne. Research reported in this publication was supported by the National Institute on Deafness and other Communication Disorders of the National Institutes of Health under award number R01DC007683 (F. H. Guenther, PI). The content is solely the responsibility of the authors and does not necessarily represent the official views of the National Institutes of Health.

## FUNDING INFORMATION

Frank H. Guenther, National Institute on Deafness and Other Communication Disorders (http://dx.doi.org/10.13039/100000055), Award ID: R01DC007683.

## AUTHOR CONTRIBUTIONS

**Matthew Masapollo**: Visualization; Formal analysis; Writing – original draft. **Jennifer Segawa**: Data curation; Formal analysis. **Deryk S. Beal**: Data curation; Formal analysis. **Jason A. Tourville**: Conceptualization; Data curation; Formal analysis, Writing – review & editing. **Alfonso Nieto-Castañón**: Formal analysis; Writing – review & editing. **Matthias Heyne**: Formal analysis. **Saul A. Frankford**: Data curation; Formal analysis. **Frank H. Guenther**: Conceptualization; Writing – review & editing.

## TECHNICAL TERMS

Basal ganglia: Set of nuclei found underneath the cortex in the medial position of the brain.
Motor sequence learning: Learning how to plan and execute an appropriate order of discrete actions.
Motor chunks: Cohesive action units composed of frequently occurring subsequences of movements.
Functional magnetic resonance imaging (fMRI): Imaging that measures changes in blood oxygenation levels that occur in response to neural firing, allowing precise localization of brain activity.
Consonant clusters: Consonant sequences at the start or end of a syllable.
Anterior insula (aINS): Brain region that plays a role in speech motor sequence learning.
Ventral premotor cortex (vPMC): Brain region that is critically important in programming speech movements.
