# Supplementary material for: Behavioral and Neural Correlates of Speech Motor Sequence Learning in Stuttering and Neurotypical Speakers: An fMRI Investigation
Source: Neurobiol Lang (Camb). 2021 Feb 1;2(1):106–37. doi: 10.1162/nol_a_00027 (PMC8294667; doi:10.1162/nol_a_00027)
Supplement: Supplementary file 1 [file nol-2-1-106-s001.pdf]

Supplementary Material for:

Neural correlates of speech motor sequence learning in stuttering vs. nonstuttering adults: an fMRI investigation

Matthew Masapollo[1], Jennifer A. Segawa[1,2], Deryk S. Beal[1,3], Matthias Heyne[1], Jason A. Tourville[1], Alfonso Nieto-Castañón[1], Saul Frankford[1], & Frank H. Guenther[1,4,5]

[1] Department of Speech, Language and Hearing Sciences, Boston University, Boston, MA 02215

[2] Departments of Neuroscience and Biology, Stonehill College, Easton, MA 02357

[3] Department of Speech-Language Pathology, University of Toronto, Toronto, ON M5G 1V7

[4] Department of Biomedical Engineering, Boston University, Boston, MA 02215

[5] Department of Radiology, Massachusetts General Hospital, Charlestown, MA 02129

S1. Given the differences in reaction time (RT) scores between the ANS and AWS groups, is the timing assumption of the sparse sampling protocol valid?

As shown in Figure 3C, the RT scores were approximately 200 ms longer in AWS than in ANS. These delayed responses were unlikely to have an effect on the obtained BOLD signal in the test phase given i.) the width of the delay of the hemodynamic response, and ii) that task performance is well under one second (as shown in Figure 3B). Nevertheless, in order to address the possibility that some trials containing "silent" stuttering blocks were included in the neuroimaging analyses, we here provide supplementary data showing the distribution of RT scores for the AWS group (Figure S2) as a whole and for each individual stuttering speaker (Figure S3) in the test phase. Critically, these plots did not reveal a bimodal distribution with one peak having frequency values clustered to the right. This finding suggests that any potential stuttering blocks or pauses were likely categorized as silent errors (sequencing subtype 4 as described; see main text for explanation) and were excluded from the imaging analyses, in which case, the BOLD responses would not have been contaminated by potential delays in RT.

S2. Figures for Supplementary Material

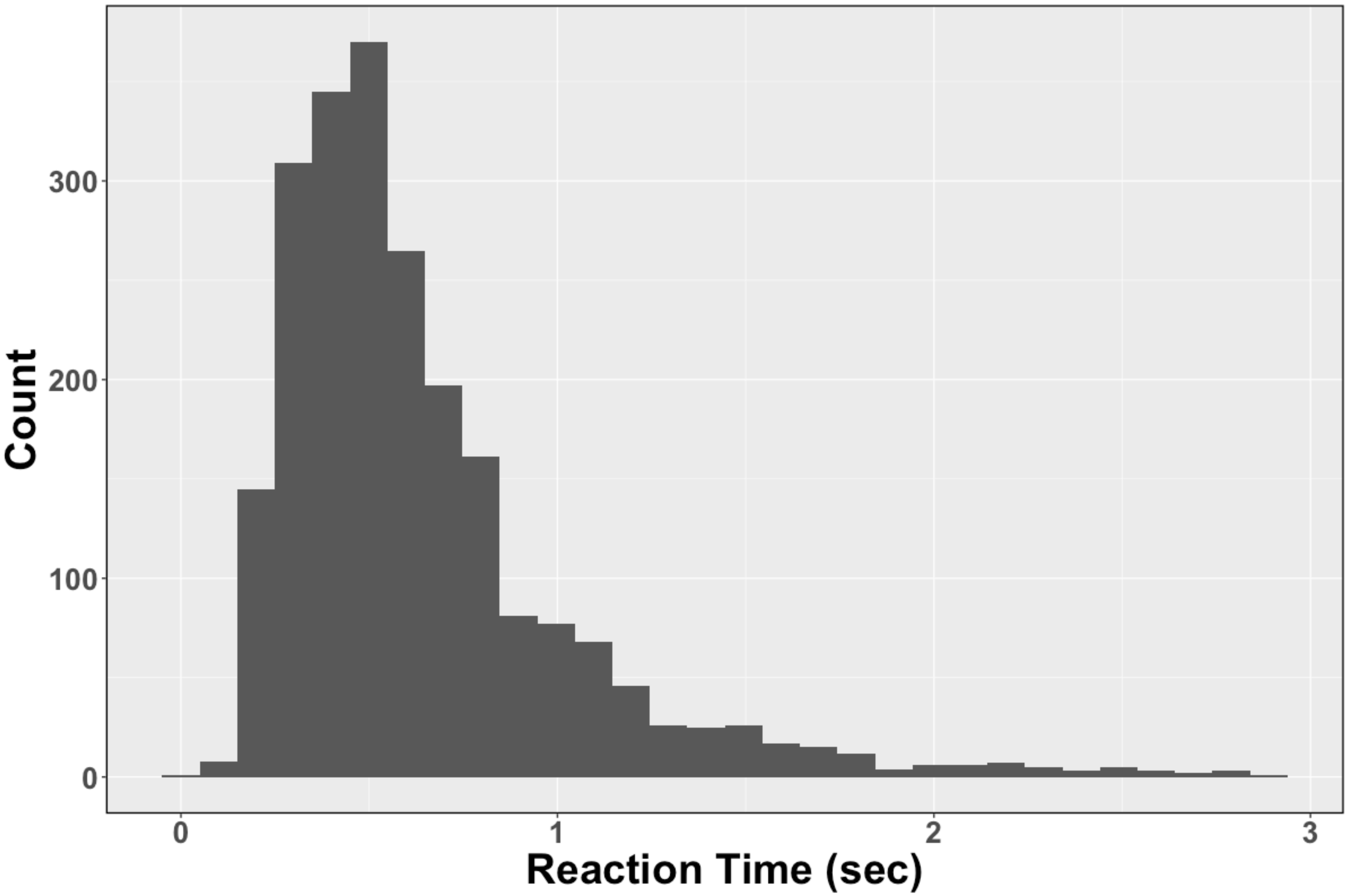

Figure S1: Histogram plots of reaction time (RT) measures (30 ms bins) for the properly sequenced productions (pooled across experimental conditions and AWS participants) during the test phase (inside the scanner). Note that the audio recording window was three seconds in duration (starting from the offset of the "go" signal).

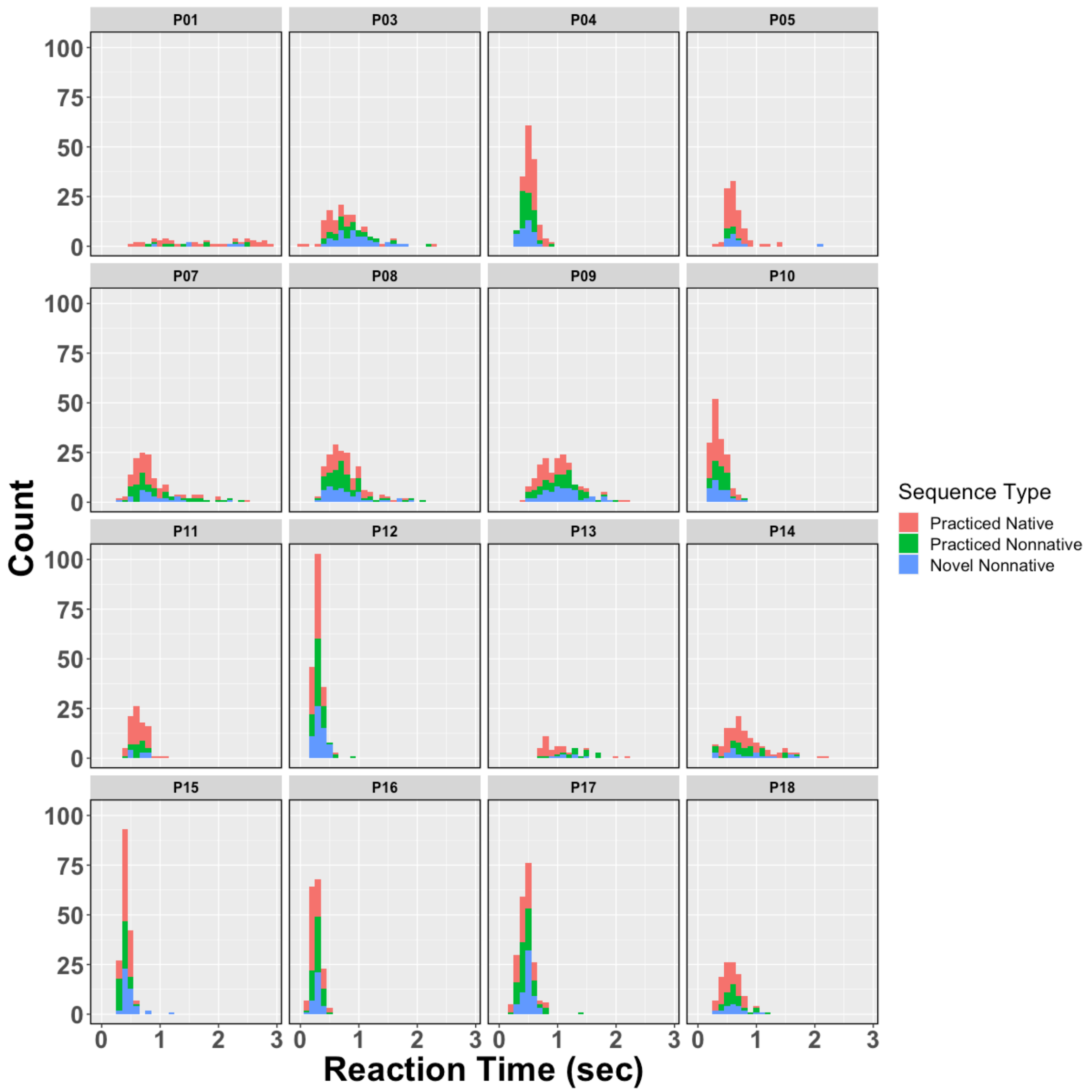

Figure S2: Histograms plots of reaction time (RT) measures (30 ms bins) for the properly sequenced productions during the test phase (inside the scanner) for each participant in the AWS group. The counts are plotted as a function of experimental condition (*practiced native* vs. *practiced nonnative* vs. *novel nonnative*) and participant (n=16). P = patient.

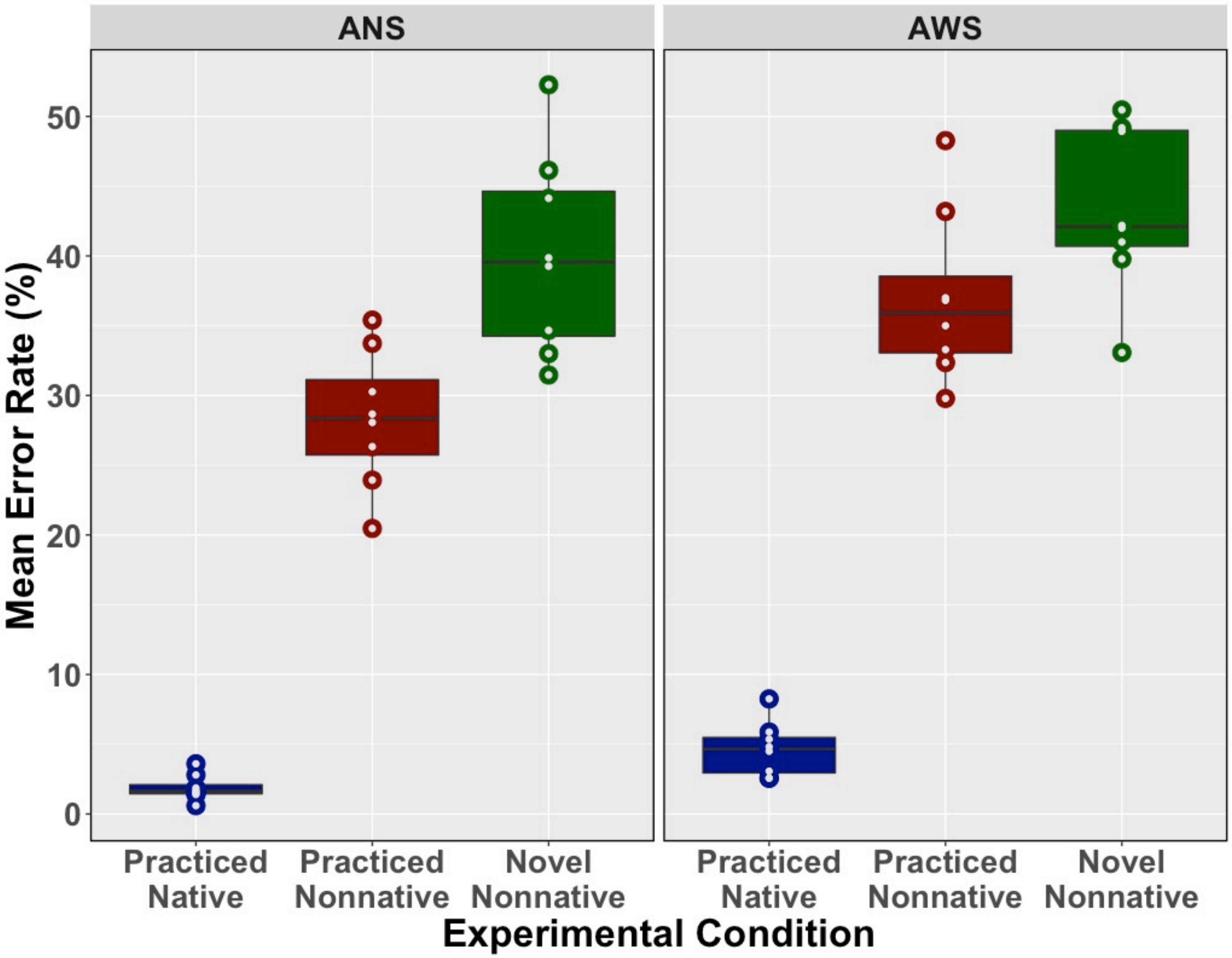

Figure S3: Boxplots of mean percentage of errors during the test phase (inside the scanner) as a function of experimental group (ANS vs. AWS) and sequence type (*practiced native* vs. *practiced non-native* vs. *novel non-native*).

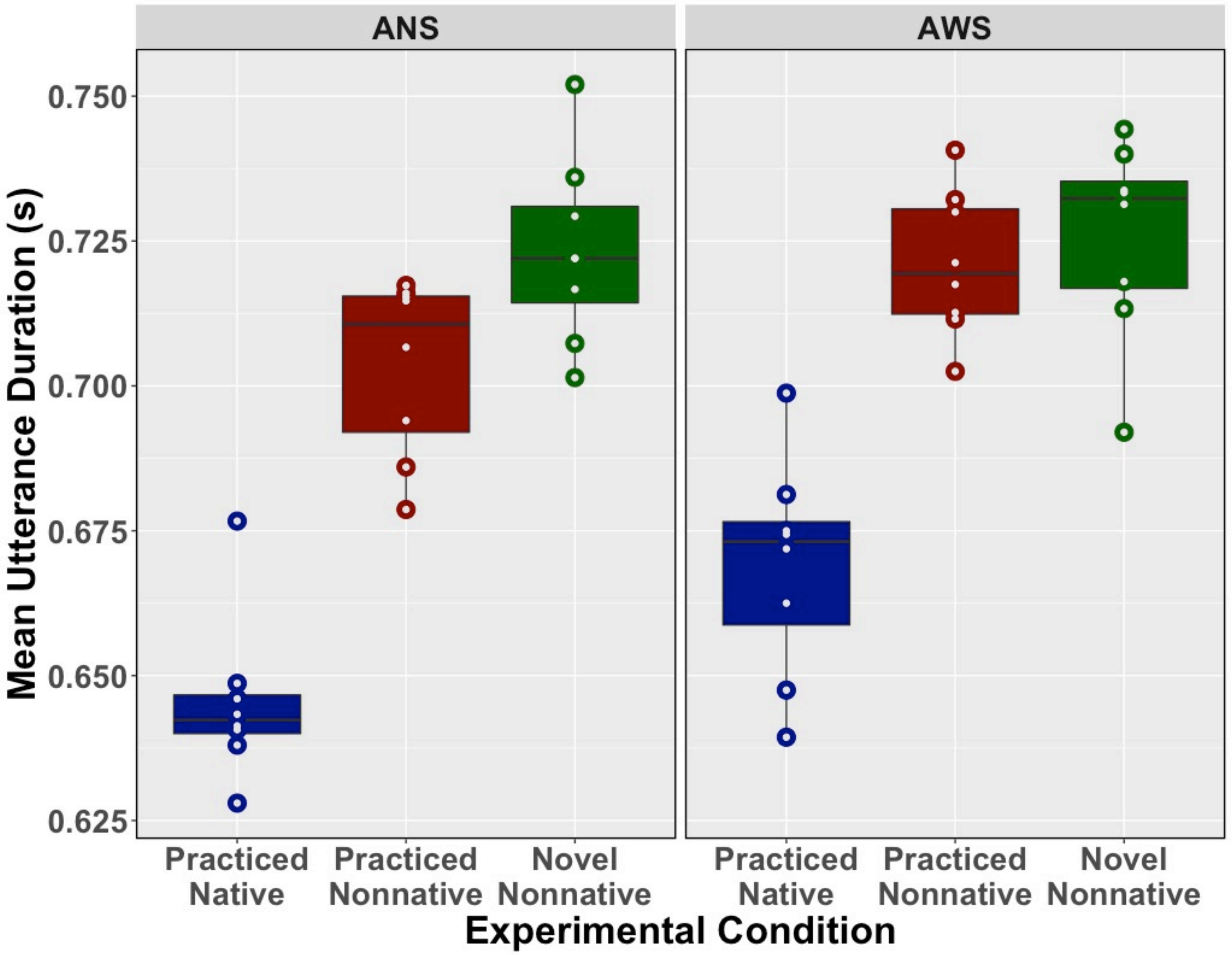

Figure S4: Boxplots of mean utterance durations (for properly sequenced productions only) during the test phase (inside the scanner) as a function of experimental group (ANS vs. AWS) and sequence type (*practiced native* vs. *practiced non-native* vs. *novel non-native*).

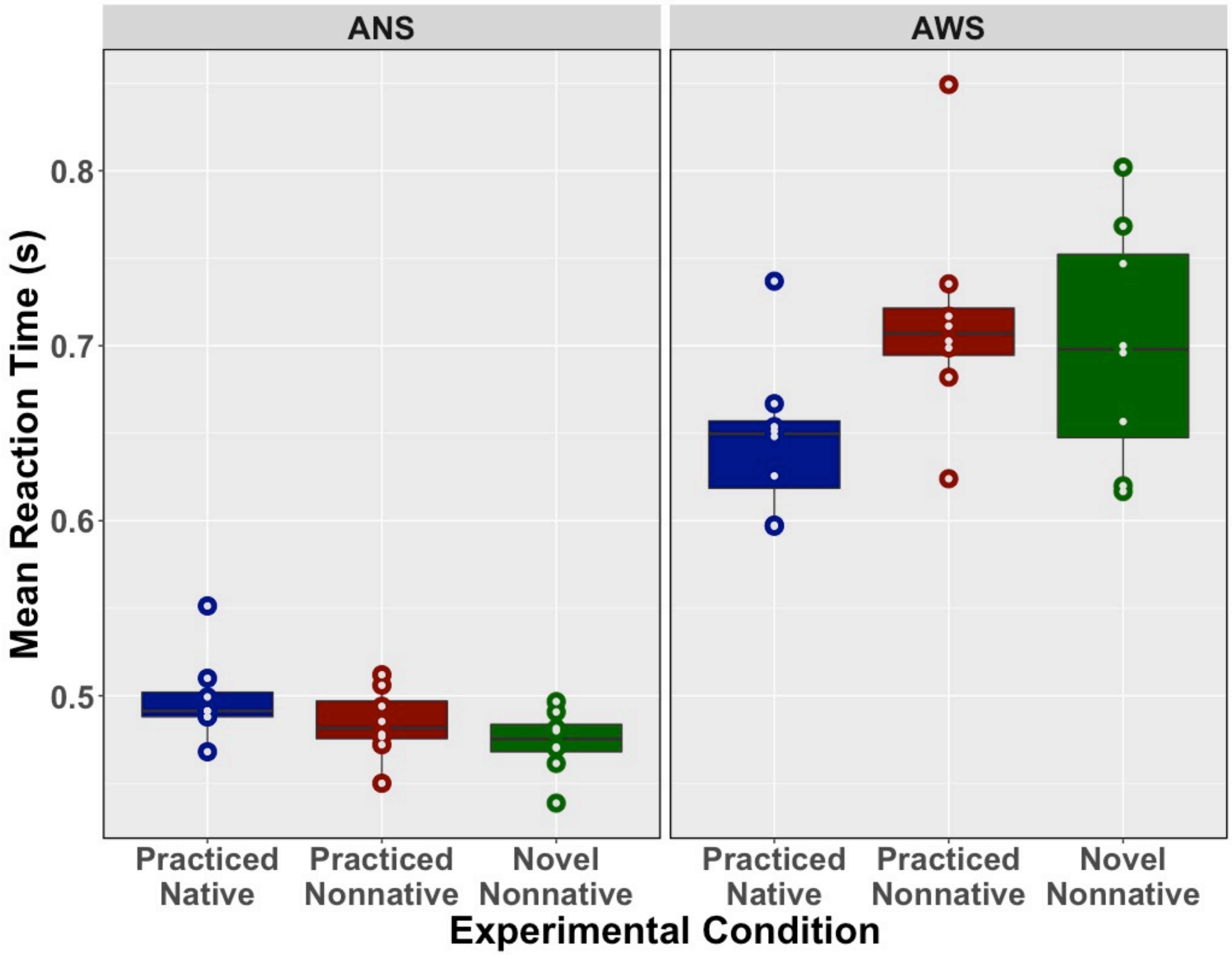

Figure S5: Boxplots of mean reaction times (for properly sequenced productions only) during the test phase (inside the scanner) as a function of experimental group (ANS vs. AWS) and sequence type (*practiced native* vs. *practiced non-native* vs. *novel non-native*).

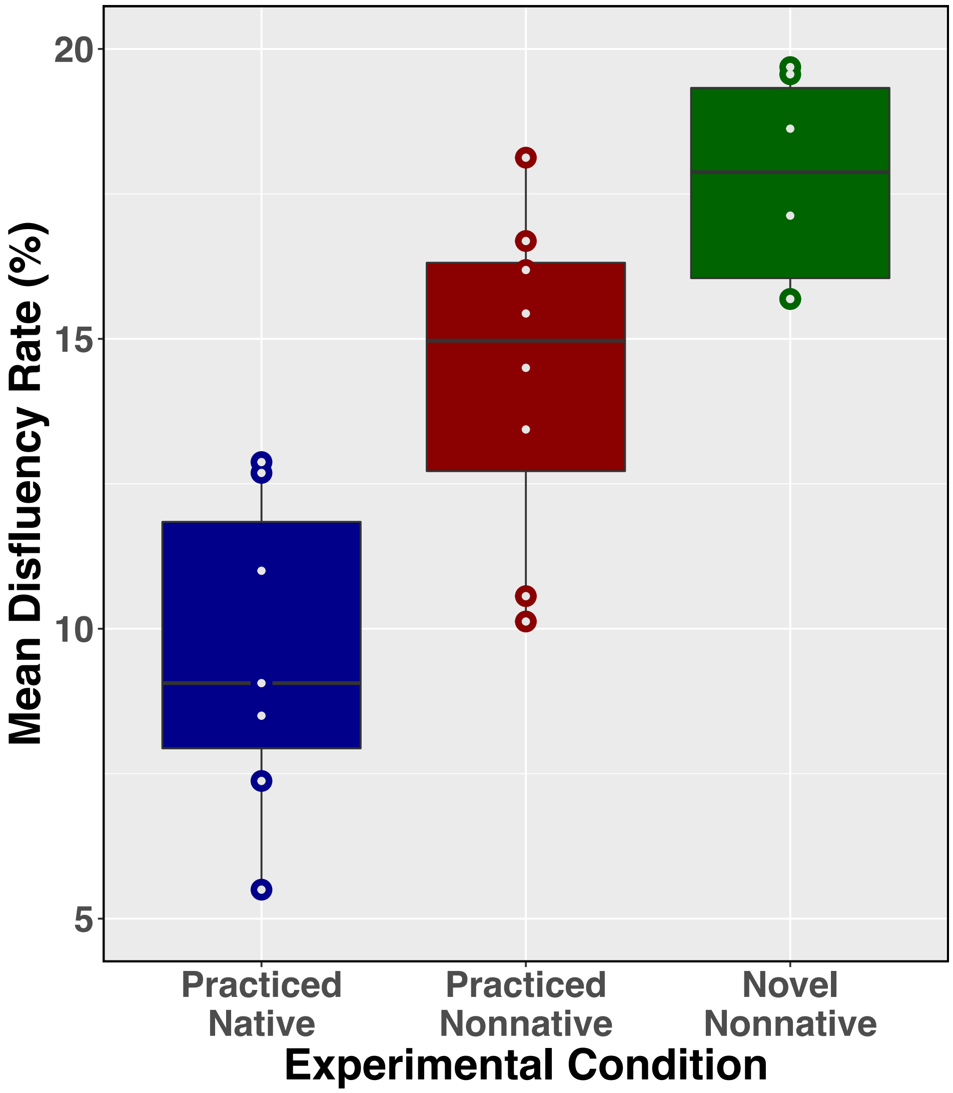

Figure S6: Boxplots of mean percentage of disfluencies during the test phase (inside the scanner) as a function of sequence type (*practiced native* vs. *practiced non-native* vs. *novel non-native*) for the AWS group only.
